# Plasmonic Supercavitation Enables Nanoparticle Photo‐Ejection Across Air/Water Interface

**DOI:** 10.1002/smsc.202500563

**Published:** 2026-03-23

**Authors:** Qiushi Zhang, Renzheng Zhang, Amartya Mandal, Dezhao Huang, Yucheng Yang, Seunghyun Moon, Jarrod Schiffbauer, Daniel A. Rhodes, James C. Hone, Matthew R. Rosenberger, Eungkyu Lee, Tengfei Luo

**Affiliations:** ^1^ Department of Aerospace and Mechanical Engineering University of Notre Dame Notre Dame Indiana USA; ^2^ School of Power and Mechanical Engineering Wuhan University Hubei China; ^3^ Department of Physical and Environmental Sciences Colorado Mesa University Grand Junction Colorado USA; ^4^ Department of Materials Science and Engineering University of Wisconsin-Madison Madison Wisconsin USA; ^5^ Department of Mechanical Engineering Columbia University New York New York USA; ^6^ Department of Electronic Engineering Kyung Hee University Yongin-si South Korea; ^7^ Department of Chemical and Biomolecular Engineering University of Notre Dame Notre Dame Indiana USA

**Keywords:** gold nanoparticles, liquid/air interface, nanobubbles, optical force, photo-ejection, plasmonic heating

## Abstract

The ability to separate miniscule solid particles (e.g., nanoparticles) from liquid is important to a wide range of applications. However, directly moving nanoparticles out of liquid is difficult as the capillary force on the nanoparticle at the liquid interface is too large for common body forces to overcome. Here, we demonstrate the ability to eject metallic nanoparticles out of liquid with a laser excitation. The laser applies an optical force on the nanoparticles to drive them toward the liquid surface. In the meantime, it intensely heats the nanoparticle to form a nanobubble encapsulating the nanoparticle (i.e., supercavitation), which achieves the liquid‐nanoparticle separation and thus eliminates the trapping force on the nanoparticle at the liquid/air interface. We show that such a mechanism can expel nanoparticles out of liquid using a transient scattering experiment, which is further confirmed by molecular dynamics simulations. We also demonstrate depositing the nanoparticles on a solid surface not in contact with the liquid. Leveraging such a feature, we show an example application where the deposited NPs are used as a template for 2D material nanotent fabrication. This study reveals an interesting fundamental mechanism to separate nanoparticles from liquid and could potentially benefit separation, nanomaterials, and biomedical applications.

## Graphical abstract

A resonant femtosecond laser drives plasmonic nanoparticles toward the air/water interface while simultaneously generating a plasmonic nanobubble, through supercavitation, that separates the nanoparticles from surrounding liquid and eliminates capillary trapping. The supercavitating nanoparticle can then be photo‐ejected into air, enabling dry, noncontact deposition.

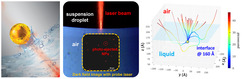

## 1 Introduction

The ability to separate miniscule solid particles from liquid is essential to a wide range of applications that need particle separation [1, 2, 3, 4, 5], concentration [6, 7, 8, 9, 10, 11], and deposition [12, 13, 14, 15, 16, 17, 18, 19, 20, 21]. For example, to study the toxicological effects of engineered nanoparticle (NP) aerosols on the human respiratory system [22, 23, 24, 25], direct deposition of solid NPs onto human cells would be ideal for in vitro experiments, but current technologies [26, 27] mostly rely on using aerosols to carry such NPs [28, 29]. Existing methods for separating solid particles from the hosting liquids, such as filtration and distillation, usually achieve solid–liquid separation by displacing liquids, i.e., passing liquids through a membrane or evaporating them into vapor. However, directly moving small particles out of liquid is more challenging, especially when their sizes approach the nanometer scale. These tiny particles can be stranded at the liquid/air interface because of the trapping force [30, 31, 32], which has led to applications such as self‐assembly [33, 34, 35, 36]. But the trapping force is so strong, depending on the size of NP and the contact angle at the interface, that common body forces are too weak to drive small particles out of the liquid. For example, for an Au NP with a diameter of 120 nm, the trapping force on it would be ~ 10^−8^ N at the water/air interface (see the Supporting Information, SI1, for calculation details) [37, 38, 39, 40]. However, body forces like optical scattering forces and magnetic forces commonly used to drive suspended NPs are many orders of magnitude smaller than the trapping force. For example, the dispersive optical scattering force on a 120‐nm‐diameter gold/silica core–shell NP is ~10^−12^ N even with a relatively high optical fluence of 9–15 mJ/cm^2^ [41, 42, 43, 44], and the magnetic force on a 10‐nm‐diameter colloidal iron oxide NP in magnetic fields with strengths of 5–15 T is only ~10^−18^ N [45]. However, if there is a way to separate surrounding liquid from the solid NP surface prior to reaching the liquid interface, there will be a chance that the NP can escape from the liquid without being stranded by the trapping force.

In our previous works, we have demonstrated that optically excited localized plasmonic heating can lead to nanoscale vapor bubbles to encapsulate the NPs, i.e., supercavitating NPs, if the optical fluence is above a certain threshold [46, 47, 48, 49, 50]. Because these nanobubbles can isolate the supercavitating NPs from surrounding liquid while the NPs are driven by optical scattering forces toward the liquid interface guided by the light [41, 43, 51, 52], they may help to realize the desired liquid‐NP separation goal. Here, we demonstrate this by directly ejecting metallic NPs out of water with a laser excitation at the surface plasmon resonance (SPR) wavelength of the NPs. The laser is shown to function as both a supercavitation exciter and an optical force provider that creates the aforementioned liquid‐NP separation and, in the meantime, drives the supercavitating NPs toward the liquid interface. We show that the optically driven supercavitating NPs can move out of the liquid as observed using a transient scattering experiment, which is also verified by molecular dynamics (MD) simulations. Our temperature field analysis using finite element thermofluidic simulations confirms that the observed ejection of NPs from liquid does not originate from boiling or evaporation. Our study reveals a novel mechanism to enable NP‐liquid separation and could potentially benefit separation, nanomaterials, and biomedical applications. As examples, we demonstrate the NP expulsion by depositing them onto a glass surface not in contact with the liquid and the fabrication of 2D materials nanotents on top of the deposited isolated single NPs.

## 2 Results and Discussion

**FIGURE 1:**
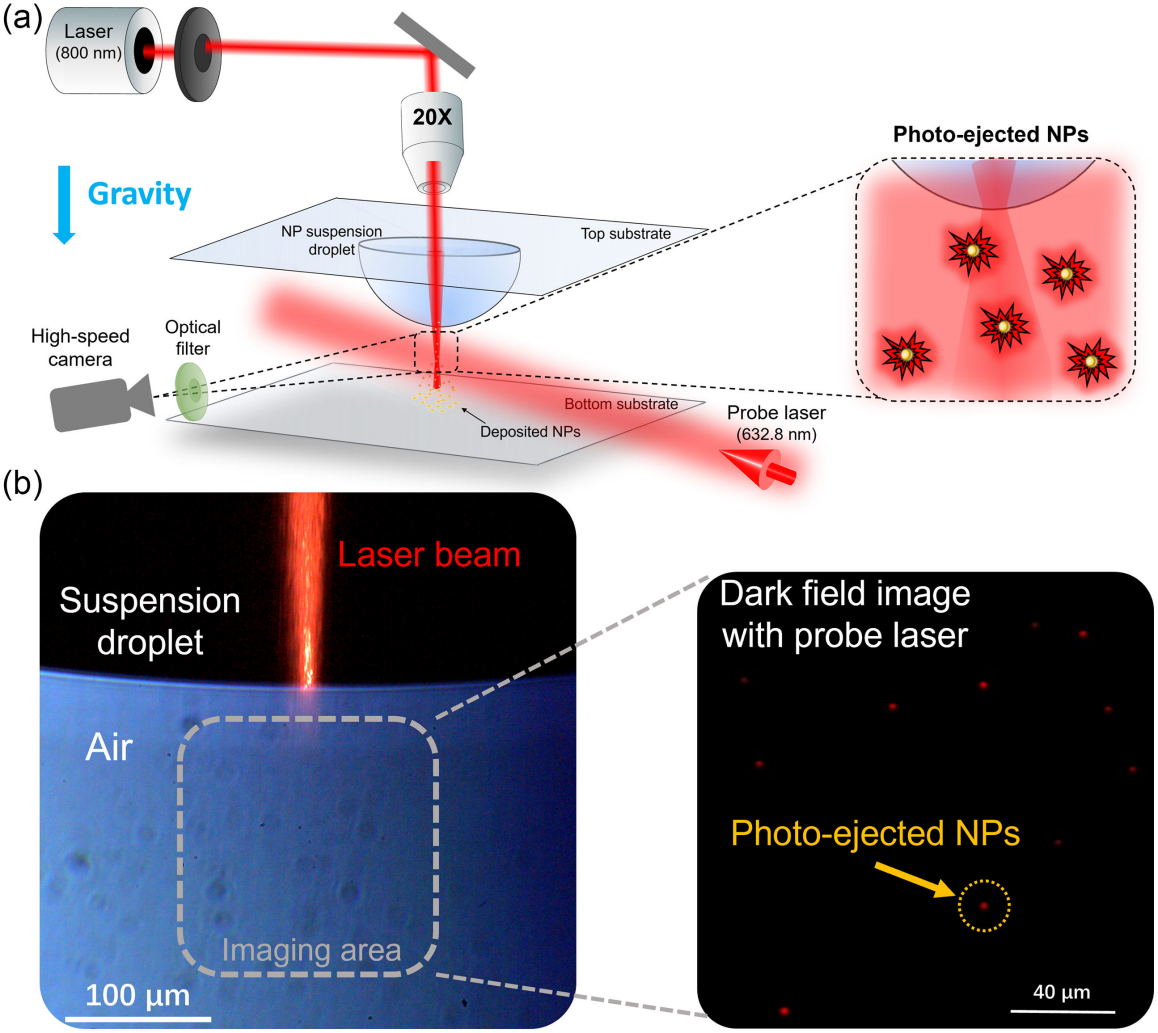
Experimental system for monitoring the photo‐ejected Au NPs across the air/liquid interface. (a) Schematic of the experimental setup to observe the photo‐ejected Au NPs into the air by a source laser at the SPR wavelength. Gravity is in the downward direction. (b) Left: bright field optical image with an LED illumination backlight showing the suspension droplet and the source laser beam focused on the air/liquid interface. Right: dark‐field optical image of the air side of air/liquid interface, which captures the photo‐ejected Au NPs. Each red glowing dot corresponds to the diffraction‐limited scattered light of the probe laser from a single Au NP [41, 51].

We first show experimentally that Au NPs (to avoid ambiguity, we clarify that throughout this work the term Au NPs specifically refers to silica–gold core–shell nanoparticles (SiO_2_@Au CSNPs)) in a suspension can be driven toward the air/liquid interface and expelled from liquid under the illumination of a laser at the SPR peak wavelength (see the Methods section for experimental setup details). The optical system is shown in Figure 1a, in which a droplet of Au NP suspension with a concentration of ~1 × 10^15^ particles/m^3^ is held by a thin glass substrate. The Au NP consists of a silica core (100 nm in diameter) and a thin Au shell (10 nm in thickness), supporting the SPR peak at ~800 nm in water. An 800‐nm femtosecond pulsed laser is focused by a 20× objective lens onto the air/liquid interface at the tip of the droplet, which is the source laser used to excite the NPs. Since the laser wavelength matches the SPR of the core–shell Au NPs in the suspension, it can intensely heat up the NPs and generate nanobubbles to encapsulate the NPs to achieve supercavitation [43, 49, 51]. The side view of the droplet is monitored by a high‐speed camera with a 10× objective lens. We use the dark‐field scattering method [41, 53] with an additional HeNe probe laser (2 mW, at the wavelength of 632.8 nm) illuminating the air side of the air/liquid interface around the focal point of the source laser to monitor the dynamics of the ejected NPs (see schematic in Figure 1a). We note that the intensity of the probe laser (0.64 W/cm^2^) is very low and thus its weak optical force should not influence the dynamics of NPs. It is also noted that we use an optical filter to block the source laser light to reach the image sensor of the camera in the dark‐field scattering measurement.

**FIGURE 3:**
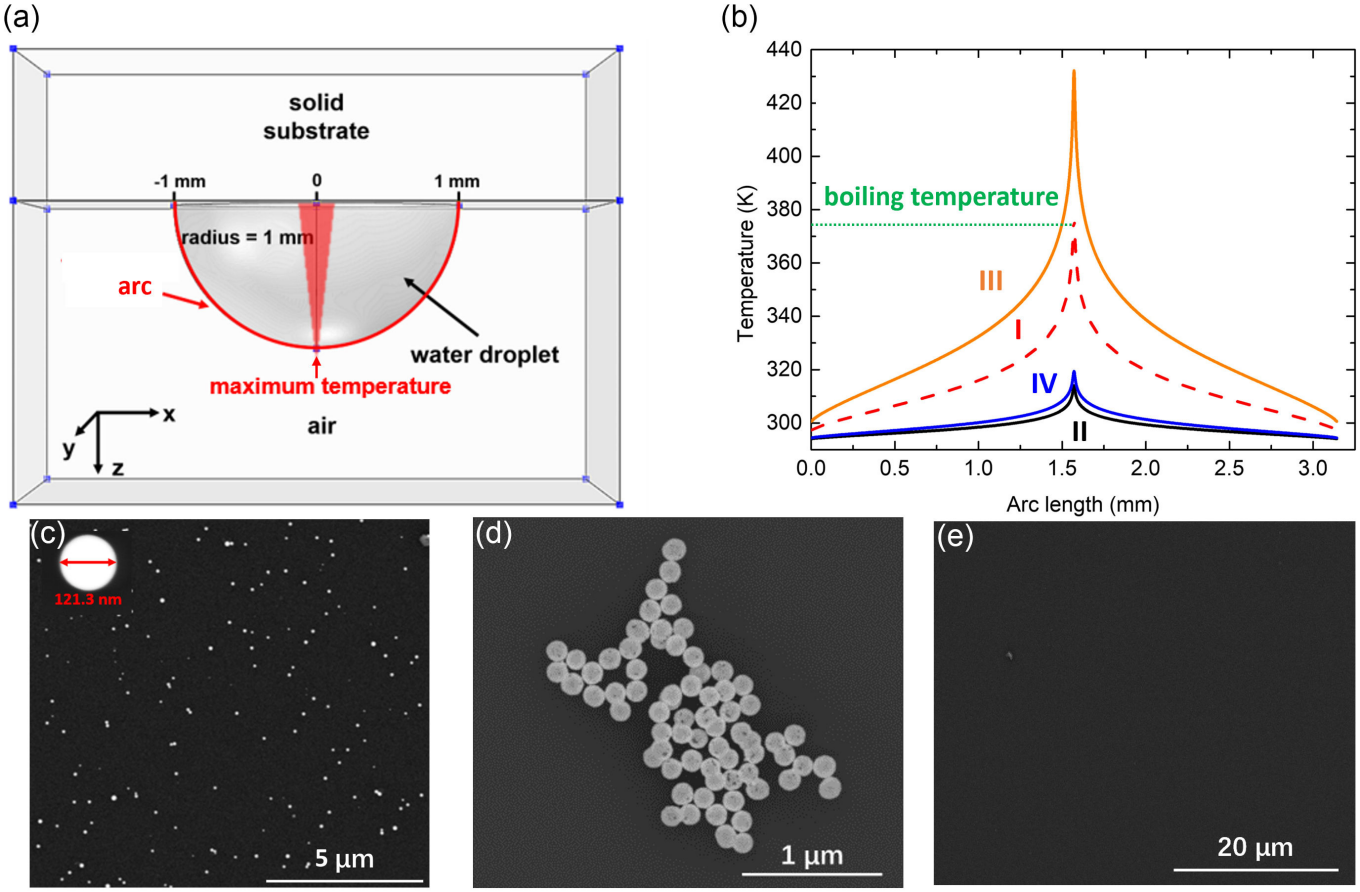
Confirmation of supercavitation as the mechanism for the laser‐driven photo‐ejection of NPs out of the liquid. (a) The geometrical configuration of the model for thermofluidic simulations to calculate the surface temperature profile of an Au NP suspension droplet under the plasmonic volumetric heating effect. The region highlighted in red indicates the region where the plasmonic volumetric heating occurs due to the illumination from a Gaussian beam. The red solid line depicts the arc line along which the surface temperature is visualized. (b) The steady‐state surface temperature profiles of the droplet along the arc line in four cases with different optical fluences and NP concentrations to realize different plasmonic heating powers. Different cases are labeled by Roman numbers I–IV. I: 22.8 mJ/cm^2^ and 2.2 × 10^15^ particles/m^3^, II: 22.8 mJ/cm^2^ and 1.0 × 10^15^ particles/m^3^, III: 22.8 mJ/cm^2^ and 3.0 × 10^15^ particles/m^3^, and IV: 5.4 mJ/cm^2^ and 2.5 × 10^15^ particles/m^3^. The water boiling temperature of 373 K is indicated by a green dashed line. The back‐scattered scanning electron microscope (SEM) images for cases (c) II, (d) III, and (e) IV are shown. The inset in (c) highlights an individual Au NP (~120 nm diameter).

As shown in Figure 1b (left), the source laser is focused on the air/liquid interface, and the air region monitored by the camera field of view is illustrated by a dashed black square. In the right panel of Figure 1b (also see the Supporting Movie, M1), where we used the source laser with an optical fluence of 22.8 mJ/cm^2^ (corresponds to the intensity of 1.8 × 10^6^ W/cm^2^) at the focal point, we can observe many red glowing dots in the dark field. These glowing dots represent the Au NPs ejected out of the suspension into air as they scatter the probe laser light in the diffraction limit [41, 43, 51, 53]. As shown later in Figure 3, these glowing dots collected by a receiving substrate are proven to be individual Au NPs. We note that these glowing dots are unlikely due to any pure water droplets, since pure water is transparent to the 800‐nm source laser or the 632.8‐nm probe laser, and there are no water interfaces to enable any optical force on the liquid itself to eject pure water droplets out of the bulk. It is also unlikely that some water droplets can come out of the bulk liquid together with photo‐ejected NPs. The Au NP surface is not functionalized by any chemical groups leading to relatively weak interfacial interactions between NP and the surrounding water molecules. Moreover, the generated nanobubble can separate the NP from its surrounding water while it is still in water [41].

**FIGURE 2:**
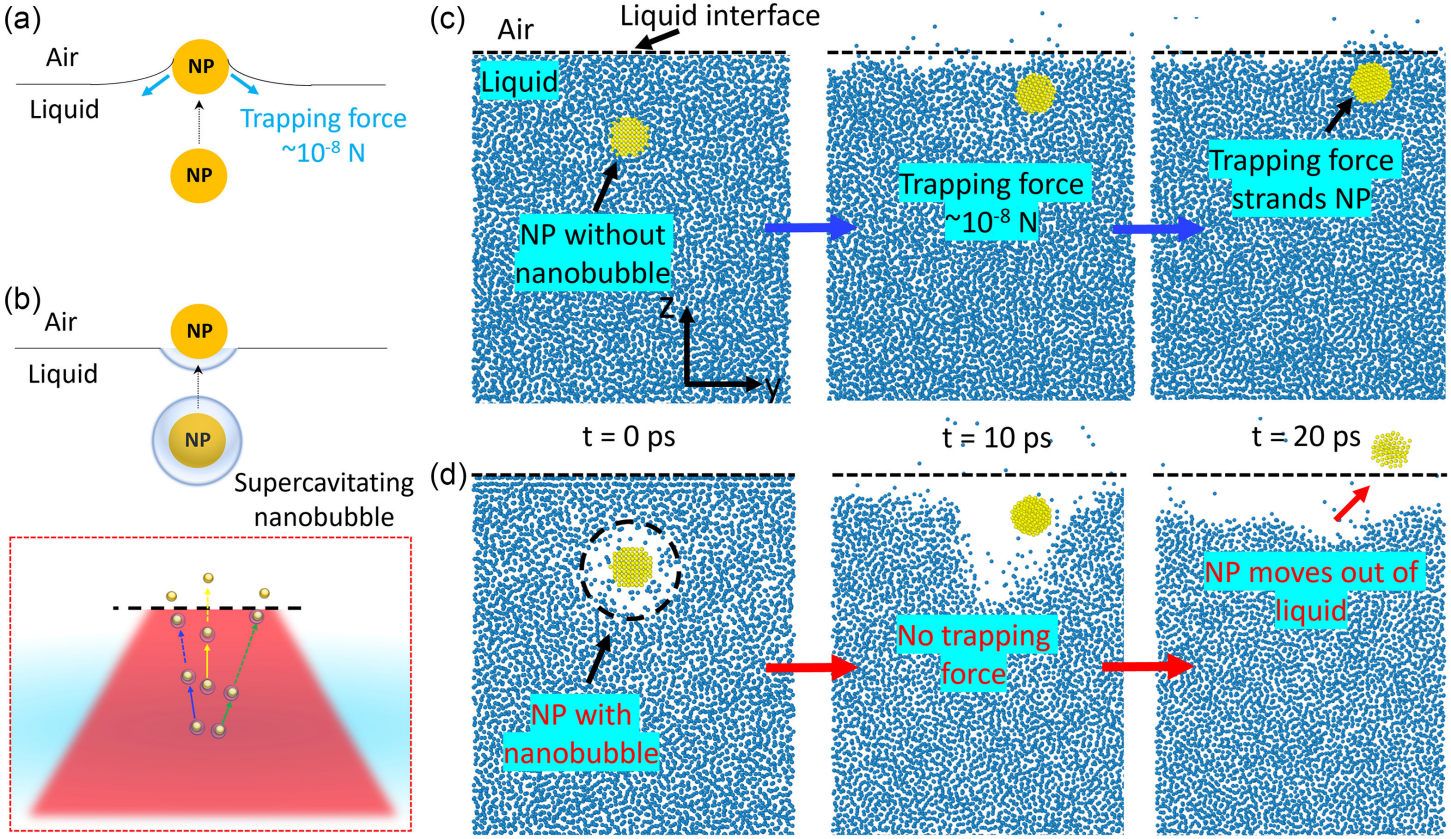
Microscopic mechanism of supercavitating NP moving out of liquid interface. Schematics showing (a) the NP stranded at the liquid/air interface due to the trapping force, but (b) with a supercavitation which separates the NP from the liquid within the liquid, the NP can pass through the interface without experiencing the trapping force at the liquid/air interface. Lower panel: The schematic of supercavitating NPs ejected out of liquid by laser. MD simulation snapshots of (c) a nonsupercavitating NP moving toward and stranded at the interface, and (d) an intensely heated NP with supercavitation moving out of liquid.

When the NP suspension is irradiated by the source laser, the optical scattering force can push the NPs toward the liquid interface [41, 51]. This force originates from the scattering of the incident photons on the NP surface, which gives a mechanical momentum to the NP in the light propagation direction [54, 55, 56]. The amplitude of this force is around 1.1 × 10^−11^ N with the highest optical fluence at the focal point of 22.8 mJ/cm^2^ used in our experiment (see the Supporting Information, SI2, for dispersive optical scattering force calculation details) [41]. However, such a force is about three orders of magnitude smaller than the trapping force (~10^−8^ N, see the Supporting Information, SI1), which can strand the NP at the liquid/air interface (Figure 2a).

For the core–shell Au NP studied in this work, when the source laser has an optical fluence above a certain threshold (~7 mJ/cm^2^) [49], the laser irradiation can lead the temperature of the irradiated NP to reach the water spinodal temperature, which is the threshold for nanobubble generation [48, 57, 58, 59]. Prior studies have shown that a moderate increase in temperature can reduce the solubility of dissolved gases in water, leading to gas exsolution into a cavity [42, 60]. Under the ultrafast, high‐fluence conditions used in our work, however, the dominant mechanism of nanobubble initiation is water vaporization. Throughout this process, the Au shell remains intact even if nanobubble encapsulated the nanoparticle for an extended period of time [51, 61], as evidenced by the undamaged shell structure shown in Figure S12C in the Supporting Information (SI8). This is because the NP is moving, and when it approaches the liquid/vapor interface within the nanobubble, more water will be evaporated, which will take away a significant amount of heat to prevent temperature getting too high [41]. The details of temperature increase corresponding to the supercavitation threshold are shown in Supporting Information (SI3). Our femto‐second pulsed laser has a fluence of 22.8 mJ/cm^2^ which is well above this threshold, and thus, a nanobubble can be generated surrounding the intensely heated plasmonic NP and encapsulate it in vapor (i.e., supercavitation) [41, 48, 49, 51, 53]. The generation of the supercavitating nanobubbles is confirmed by the transient scattering pump‐probe experiments as shown in the Supporting Information (SI4), Supporting Movies M2 and M3, and our previous works using the same laser setup and parameters as in this work [41, 62].

While the optical dispersive force from the source laser can drive the NP toward the liquid/air interface, supercavitation separates the solid NP from the liquid via a thermally induced phase change process [50, 63, 64, 65] before the NP reaches the liquid/air interface, which in turn eliminates the need to overcome the trapping force at the liquid/air interface (Figure 2b). We note that the size of the nanobubble, which is estimated to be in the order of hundreds of nm in radius [41, 49], or the nature of the nanobubble (vapor vs. gas) [50, 66] does not influence the photo‐ejection mechanism as long as the supercavitation serves the purpose of separating the NP from the surrounding liquid.

To confirm this hypothesis, we perform a series of MD simulations of a solid NP immersed in liquid moving toward the liquid interface without and with thermally induced supercavitation (see the Methods section for MD simulation details). We simulate an Au NP with a radius of 1 nm immersed in liquid argon, which has a free surface (Figure 2c,d). In one case, both the NP and the liquid are kept at 90 K, and thus no supercavitation is present. In the second case, the NP is heated to and maintained at 1000 K to excite a nanobubble encapsulating the NP [67]. Because the nanobubble lifetime is much longer than the laser pulse interval in the experiment [68], the bubble will not collapse before the next pulse arrives to maintain the high temperature of the NP. Hence, it is reasonable to keep the NP at a high temperature in the MD to mimic the experiment. We note that as long as the supercavitation can be generated, the exact heating temperature of the NP will not influence the conclusion of the simulations. As shown in Figure 2c, the NP is stranded by the liquid interface due to the trapping force (see also the Supporting Movie M4). However, when a supercavitation is generated to encapsulate the NP, the NP can move across the liquid interface without any impedance (Figure 2d and the Supporting Movie M5). In this case, the NP‐liquid separation is achieved when the nanobubble is generated, and when the NP approaches the liquid interface, there is no longer a trapping force holding back the NP from moving out of the liquid. We note that while these simulations are on a simplified model system of NP‐in‐liquid‐argon, the observation should be generally applicable to verify our hypothesis. There are many ways real systems can be more complicated than the model simulated here. For example, strong hydrogen bonds may exist between NP and liquid molecules depending on the NP surface functionalization. We can mimic such stronger interfacial interactions by tuning up the energy constant of the interaction between the NP and the liquid in our simulations. However, after increasing the energy constant of the L‐J potential by 10 times, the observation stays the same, i.e., the NP with supercavitation can still escape from the liquid and move into the air (see the Supporting Information, SI5). We also validated our findings with more realistic water models, SPC/E and TIP3P (see Methods section for details), where similar NP photo‐ejection phenomena were observed (see the Supporting Information, SI5, for the simulation results). Although the simulated particle (≈1 nm) is far smaller than the 120‐nm core–shell NP used experimentally, prior MD studies [50, 69] have consistently shown that the radius of the laser‐induced vapor layer is on the same order of the particle radius for both Lennard‐Jones fluids and explicit water. Pump–probe experiments [70] report the same scaling trend, indicating that bubble size grows roughly linearly with particle diameter. Thus, the decisive factor is not the absolute size but the formation of a nanoscopic vapor layer that decouples the solid from the liquid. Once this separation exists, the trapping force vanishes and the NP can traverse the interface, regardless of scale. Therefore, these results confirm that the thermally excited supercavitation enables the light to separate the NP from liquid and eject it across the air/liquid interface into the air.

To further confirm that the observed laser‐driven photo‐ejection of Au NPs is due to the supercavitation, we need to exclude the effect of evaporation or boiling of the NP suspension due to the laser‐induced volumetric heating [44], which may also spread NPs into air from the suspension droplet (Figure 1). We perform thermofluidic simulations using the finite element method to calculate the steady‐state temperature profile of a liquid droplet subject to laser heating due to the light absorption of the suspended plasmonic NPs. The simulation model is shown in Figure 3a, in which a water hemisphere with a radius of 1 mm is used to simulate the suspension droplet in the experiment. The plasmonic volumetric heating following a Gaussian distribution is the heating source in the system with the highest intensity located at the tip of the hemisphere, which mimics the focused laser beam (Gaussian beam) in our experiment [43, 44, 51]. The heating power of the system is determined by the laser optical fluence and NP concentration in the suspension. More details of the simulations can be found in the Supporting Information, SI6. Since the highest temperature should occur at the laser focal spot, i.e., *t*he tip of the droplet, we plot the steady‐state surface temperature profile of the hemisphere along the red line indicated in the model shown in Figure 3a to investigate the potential evaporation or boiling effect.

Four cases with different heating powers are studied by changing the optical fluence and NP concentration. The temperature profiles of the four cases are plotted in Figure 3b. As expected, all the four cases have a symmetric surface temperature profile with the maxima located in the middle corresponding to the laser focal point. In case I (red dash line in Figure 3b), where the optical fluence and NP concentration are respectively 22.8 mJ/cm^2^ and 2.2 × 10^15^ particles/m^3^, the maximum temperature is ~373 K (100°C), which is the water boiling temperature. We note that the pressure difference induced by the droplet interface curvature, the Laplace pressure, on water boiling point is negligible for our millimeter‐size droplet (see the Supporting Information, SI7, for details). Case II (black solid line in Figure 3b) uses the same peak optical fluence (22.8 mJ/cm^2^) but a lower NP concentration of 1.0 × 10^15^ particles/m^3^. These parameters are the same as we used in the experiment to visualize the NP ejection in Figure 1. Because of the lower heating power in case II, the maximum temperature is only ~50°C—well below the water boiling threshold. This means the observed Au NPs in air we previously imaged in Figure 1b (right) are not the results of bulk liquid boiling that might spit out small suspension droplets. To further investigate the Au NP photo‐ejection mechanism, we placed a thin glass slide at a distance of 0.5 mm away from the tip of the suspension droplet (see Figure 1a), so that the NPs expelled out of the liquid by the laser can be deposited on the slide (see the Methods section for experimental details) and then can be visualized using SEM. As the back‐scattered SEM image shown in Figure 3c (also see the dark‐field optical images in the Supporting Information, SI8), a large number of individual Au NPs are deposited on the glass slide. It is worth noting that there might be a few dimers present on the substrate, possibly resulting from the hybridization of the NPs with defects on the stabilizing surface ligands, which could occur within the suspension or on the depositing substrate. The inset in Figure 3c highlights a zoomed view of an individual NP, whose diameter is ~ 120 nm, and elemental composition (EDX result in the Supporting Information, SI8) confirms that these are Au NPs from the suspension droplet.

As a comparison to case II, which has a maximum surface temperature below the boiling point, we study another case, case III, where the optical fluence is kept at 22.8 mJ/cm^2^, but the NP concentration is increased to 3.0 × 10^15^ particles/m^3^. The resulted higher heating power leads to a maximum temperature of ~160°C, well above the water boiling temperature of 100°C (orange line in Figure 3b). In the experiment with the parameters corresponding to this case, we can also observe NPs deposited on the glass slide, but different from the scattered‐distributed individual NPs in case II, we find many small clusters of NPs deposited on glass slide in case III. The back‐scattered SEM image in Figure 3d shows an example of such clusters containing around ~ 100 NPs. It is worth noting that in our previous works [41, 51], where the same laser and NP suspension were used, we found that supercavitating NPs were mostly isolated particles in the suspension instead of clusters of NPs collectively encapsulated by a large bubble. Therefore, when they are ejected out of the liquid interface, they should be deposited individually as we see in case II (Figure 3c). However, when the heating power is sufficiently high to cause the droplet to boil as in case III, there can be tiny droplets splashing out of the suspension droplet. These tiny droplets can contain many NPs, and when they reach the glass slide and dry out, they can leave small clusters of NPs on the glass slide through a contact line deposition mechanism [12, 71].

While the maximum temperature in case II (~50°C) is not sufficient for boiling, the elevated temperature can increase evaporation. To ensure such enhanced evaporation cannot cause NPs to escape from the liquid droplet, we study another comparison case IV, where the peak optical fluence is reduced to 5.4 mJ/cm^2^ but the NP concentration is increased to 2.5 × 10^15^ particles/m^3^, to still achieve the similar surface temperature as case II (blue solid line Figure 3b). We note that the peak optical fluence of 5.4 mJ/cm^2^ is lower than the supercavitation threshold, 7 mJ/cm^2^, as reported previously [49]. In this case IV, there is no NP deposited on the glass slide that can be observed in the back‐scattered SEM (Figure 3e). This further confirms that supercavitation is the prerequisite for the laser to expel the NPs out of liquid.

**FIGURE 4:**
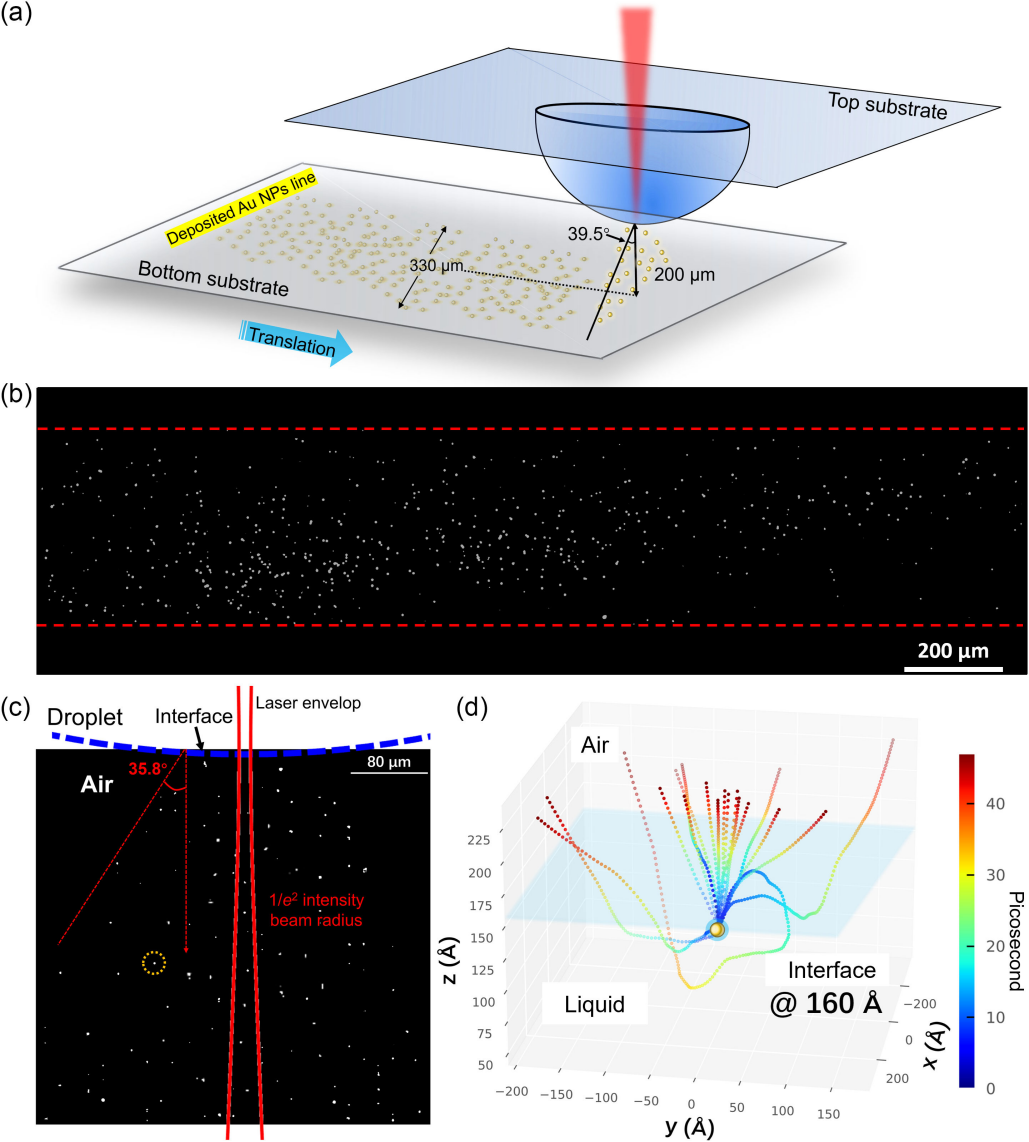
NP pattern writing and ejection angle analysis. (a) Schematic of the NP‐line deposition experiment. (b) The back‐scattered SEM image of the line of deposited Au NPs on the substrate by the laser with a fluence of 22.8 mJ/cm^2^ and a NP concentration of 1.0 × 10^15^ particles/m^3^ in the droplet (also refer to the optical image in the Supporting Information, SI8). The red dashed lines draw the approximated boundaries of the deposited line to guide the eyes. (c) The dark‐field microscopy spatial distribution of the NPs photo‐ejected into the air region (grayscale). The bright spots (circled in yellow) represent the locations of NPs ejected into air. The calculated 1/e^2^ intensity profile of the Gaussian source laser beam is overlapped in the image. (d) The 3D MD‐simulated trajectories of 18 different supercavitating NPs moving from liquid into air (the interface is located at 160 Å).

A potential application of this technique using photo‐ejected NPs out of liquid is writing NP patterns on the surfaces which are sensitive to liquids. Although different NP deposition methods exist [15, 43, 72], most of them are based on wet processes, which require immersing the surfaces in liquids that contain the target NPs. However, this requirement can induce problems for sensitive materials or devices, such as metals or electronics that cannot be immersed in liquid solvents due to the concerns like corrosion or short‐circuiting. Here, we use this photo‐ejection enabled NP deposition technique to demonstrate the writing of a ~2 mm‐long line of individual Au NPs on a glass substrate by translating the bottom substrate linearly while performing the deposition (see the schematic of setup in Figure 4a and SEM image in Figure 4b). The distance between the bottom glass substrate and the tip of the droplet is ~200 μm. Under this distance and NP concentration of 1.0 × 10^15^ particles/m^3^, the deposition rate is around a few hundreds of NPs per minute. It is interesting to see that the deposited NPs are spread out across the width of the written line with a range of ~330 μm as can be seen in Figure 4b. The density of deposited NP increases closer to the center of the written line. We believe the spread of NP is because the NPs are leaving the liquid interface over a range of angles and locations with respect to the laser beam axis. As shown in Figure S11, certain ejected NPs can travel significantly farther and be deposited at locations ~500 µm away from the center. Using the dimensions shown in Figure 4a, we can estimate that the spread angle of the NP deposition is ~ 39.5°, which is similar to the estimated angle (35.8°) from the dark‐field microscopy which tracks the locations of the NPs ejected out of the liquid (Figure 4c).

The 1/$e^{2}$ diameter of the Gaussian source laser beam at the focal point, which is at the tip of the droplet, is ~12 μm, and the spread of the laser beam envelop after exiting the liquid interface (see the Supporting Information, SI9, for calculation details) is much smaller than the observed spread of the trajectories of NPs coming out of the liquid (Figure 4c). Thus, the cause of the spread in the NP exiting angle is unlikely to be the divergence of the laser beam.

We believe the spread angle is due to the stochastic nature of the nanobubble formation and the relative position of NP inside the nanobubble. It is known that the nanobubble formation is stochastic as the nucleation of the vapor bubble depends on the local temperature profile and the defects of the NP surface [50, 73]. This can lead to the randomness in the fluidic forces on the NP, which can in turn change its moving direction [67, 74, 75, 76]. Moreover, the relative position of NP inside the nanobubble is also stochastic, undergoing ballistic Brownian motion within the bubble [67], and the randomness of the NP‐bubble position can lead to different optical configurations and thus redirect the dispersive optical scattering force direction on the NP [41, 53]. As seen in the Supporting Movie M6, which shows the NP movement across the laser focal plane when the laser is focused in a liquid, supercavitating NPs can indeed move with some randomness in their directions, although in the long spatial range, they still stay within the laser envelope, which is understandable as their movements are driven by the laser. Therefore, when the supercavitating NPs leave the liquid interface at the tip of the suspension droplet, where the laser is focused on, the randomness in NP movement allows them to fly out in directions that are not aligned with the laser beam axis (see the schematic in Figure 2b).

Similar phenomena can also be observed in the MD simulations of the ejection process where we launch 18 supercavitating NPs (diameter = 1 nm) one‐by‐one toward the liquid interface from a distance (~4 nm) away from the interface (Figure 4d). We find that each of the NPs comes out of the liquid interface at a different angle and a different location (also see the Supporting Movies, M7 and M8). The MD‐simulated spread angles of these 18 NPs are plotted in the Supporting Information, SI10. These simulations show that the stochastic nature of the NP movement can lead to the spread of the ejected NPs.

**FIGURE 5:**
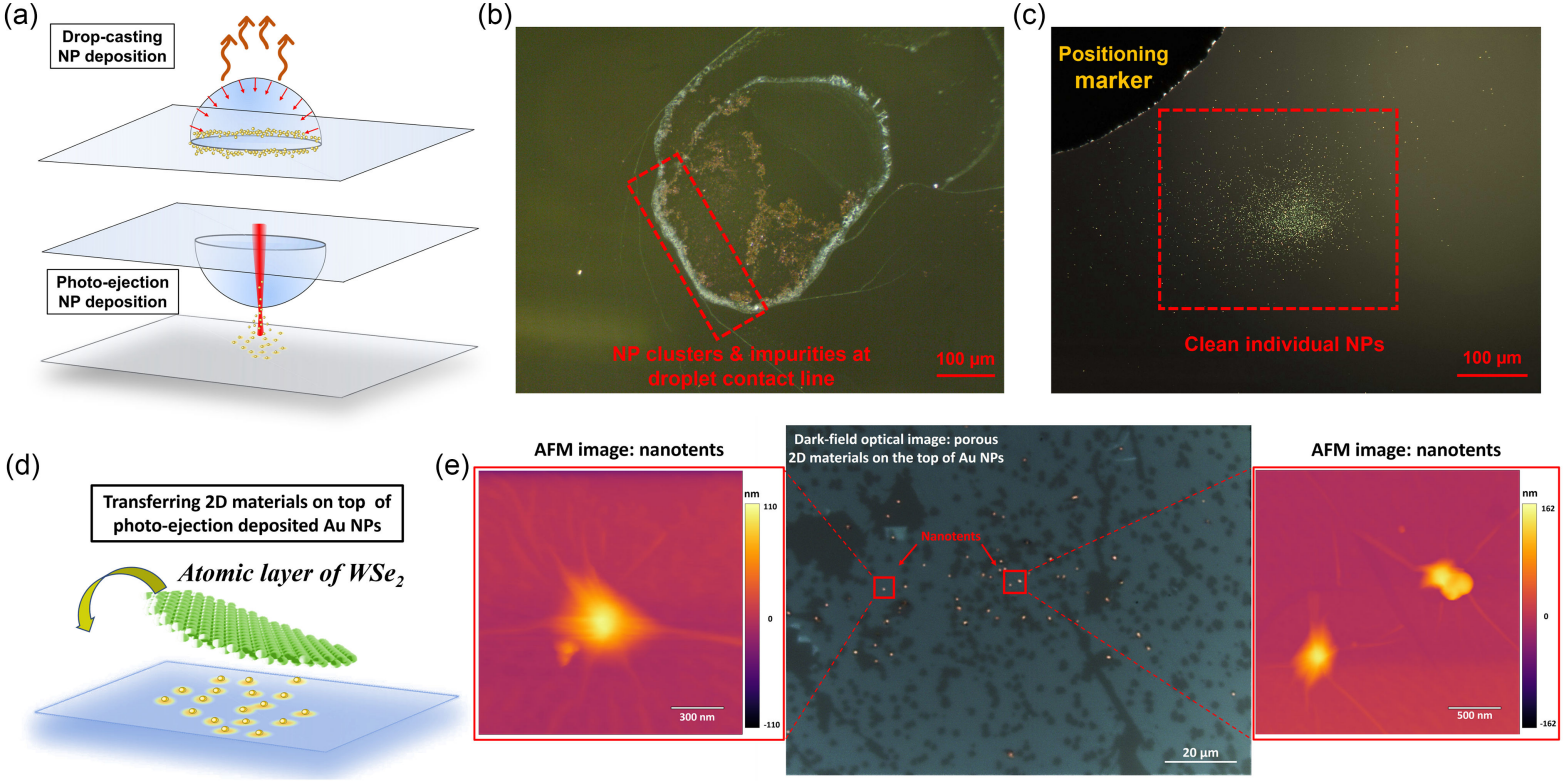
2D material nanotent fabrication with photo‐ejection NP deposition. (a) Schematic representation of the drop‐casting (upper) and photo‐ejection (lower) methods for NP deposition. Optical images displaying the sites of NP deposition using the drop‐casting (b) and photo‐ejection (c) methods. (d) Schematic of nanotent fabrication involving the transfer of an atomic layer of WSe_2_ onto a quartz substrate using photo‐ejection‐deposited Au NPs. (e) Middle: Optical image showing the WSe_2_ layer on top of the deposited Au NPs (depicted as glowing dots). Circled in red are examples of formed nanotents, accompanied by corresponding high‐resolution AFM images in the left and right panels. Scale bars indicate the magnitudes of height in the AFM images.

Photo‐ejection NP deposition offers an additional advantage by ensuring a cleaner deposition site compared to certain conventional NP deposition methods that necessitate contact between the depositing substrate and the NP suspension. For instance, one common such method is drop‐casting, which relies on the evaporation of liquid to deposit NPs through the coffee‐ring effect (Figure 5a) [71]. However, during the evaporation process, impurities present in the suspension may also accumulate at the NP deposition site. This can be observed in Figure 5b, where a significant number of impurities remain alongside the deposited NPs at the contact line of the dried‐out suspension drop. In contrast, the photo‐ejection method shows a much cleaner deposition site since the deposition substrate remains free from any contact with the suspension (as shown in Figure 5c). Consequently, the photo‐ejection deposition method reduces the need for stringent preparation steps to achieve a highly pure NP suspension for NP deposition.

The deposition of individually isolated NPs on a substrate using the photo‐ejection method provides an excellent platform for inducing nanotent deformations in 2D materials [77, 78, 79, 80]. These localized out‐of‐plane deformations generate nonuniform and self‐sustained in‐plane strain, which is crucial for strain engineering in 2D materials with the potential for quantum information technologies [81, 82, 83]. As an example for potential applications, we present the fabrication of nanotents by transferring a single atomic layer of WSe2 onto a quartz substrate with photo‐ejection‐deposited Au NPs (refer to Figure 5d and the Methods section for experimental details). Despite the presence of defects in the WSe_2_ layer, as evident from the dark‐field optical image in Figure 5e (middle panel), which is a common issue in 2D material fabrication, we can readily observe NPs covered by the WSe2 layer. High‐resolution atomic force microscopy (AFM) allows clear visualization of the nanotents formed by the WSe_2_ layer on individually isolated NPs, as depicted in Figure 5e. The photo‐ejection method enables the deposition of a large number of individual NPs, spanning a substantial area of several hundred micrometers. Consequently, the fabrication of nanotents no longer necessitates large‐scale defect‐free 2D materials or precise manipulation of 2D materials. While NP deposition serves as an illustrative example, the photo‐ejection mechanism of NPs holds potential for other applications, such as moving NPs across interfaces between immiscible liquids and selectively depositing different plasmonic NPs using lasers with the wavelengths matching their specific SPR peaks.

## 3 Conclusion

In conclusion, we have demonstrated laser‐driven photo‐ejection of plasmonic NPs from liquid, which would not have been possible using conventional body forces. The laser with a wavelength at the SPR peak of the NPs can induce an intense heating effect and form supercavitations. This process achieves the liquid‐NP separation via a thermally induced phase change mechanism, and thus when the NPs are driven to the liquid‐free interface by the optical scattering force, they can move out of liquid without being stranded by the trapping force on the NP surface. Using finite element thermofluidic simulations, we prove that the observed NPs ejected out of liquid do not originate from any boiling or evaporation effect, confirming the supercavitation as the key. The NPs expelled out of liquid by the laser can be deposited on a substrate as individuals, which is an ideal templet to induce local deformation into 2D materials, i.e., nanotents. By visualizing the deposited NPs, we observed a spreading angle larger than the divergence of the Gaussian laser beam, and this is attributed to the stochastic nature of nanobubble formation and the relative position of NP inside the nanobubble which result in fluctuations in the NP moving direction. This study reveals an interesting mechanism to separate NPs from a suspending liquid environment and could potentially lead to applications that utilize or need NP dry deposition and separation.

## 4 Methods

### 4.1 Optical System to Image the Photo‐Ejected Au NPs out of Liquid Into Air

Figure 1a schematically shows the experimental setup used to probe the Au NPs ejected by laser from liquid into air. A femtosecond mode‐locked monochromatic pulsed source laser (repetition rate of 80.7 MHz and pulse duration of 100 fs) from a Ti:sapphire crystal in an optical cavity (Spectra Physics, Tsunami) is directed to an Au NP suspension droplet. The center wavelength of the laser is 800.32 nm with a full‐width‐half‐maximum of ~10.5 nm. The laser beam is focused by a 20× objective lens (Edmund Optics, numerical aperture = 0.42, focal length = 10 mm) onto the tip of the hemispherical droplet. In this work, we use optical fluence (F) to quantify the energy of the pulsed laser, which can be calculated from either the time‐averaged laser power ($P_{L}$), which is measured by an optical power meter (Thorlabs PM130D), or power intensity (I) by:

(1) $$F=\frac{2\times P_{L}}{\pi \times {w_{0}}^{2}}\times \frac{1}{f}=I\times \frac{1}{f}$$

where $w_{0}=6\;\text{µ}m$ is the 1*/e* ^2^ Gaussian laser beam radius at the focal point, which is determined from a scanning‐slit optical beam profiler (Thorlabs BP209), and f is the repetition rate of the pulsed laser. The peak optical fluence at the focal point can be tuned continuously from 0.22 to 22.8 mJ/cm^2^ using a continuously variable metallic neutral density filter (NDC‐25C‐4M, Thorlabs). An optical shutter controls the on/off of the source laser. A high‐speed digital camera (HX‐7, NAC) with a 10× objective lens (Edmund Optics) is used to record the side view. A white LED (300 lumens) illumination source is used for the bright‐field imaging, and a coherent probe laser source with a wavelength of 632.8 nm (HeNe, 2 mW, Thorlabs) is used to image the NPs ejected from liquid in the dark‐field setting. The probe laser beam is directed to have an angle, ~90°, to the imaging axis of the high‐speed camera (see Figure 1a) so that the probe laser cannot be seen by the camera directly, but the camera can capture the scattered probe laser light from the Au NPs ejected from liquid into air in the dark‐field setting. We note that an optical filter is placed in front of the camera to filter out the source laser light (Figure 1a). The spatial distribution of NPs ejected into air shown in Figure 4c is obtained by overlapping each frame of the captured video (similar to the Supporting Movie M1) in 10 s with a customized image processing code in MATLAB.

### 4.2 Substrate Preparation and Au NP Suspension

Both the top and bottom substrates in Figure 1a are made of glass. Before attaching the Au NP suspension droplet, the substrates were sequentially cleaned with acetone, isopropyl alcohol, ethanol, and deionized water in an ultrasonic bath (PH30 Digital Ultrasonic Cleaner, Elma) and dried in a vacuum chamber (BACOENG). The Au NP (AuroShell, Nanospectra Biosciences, Inc.) used in this work consists of a silica core and an Au shell. The solvent for the Au NP suspension is deionized water produced by the Barnstead NanoPure Diamond system with a purity of 18 MOhm. These core–shell Au NPs have the near‐infrared SPR wavelength of 780–800 nm. This near‐infrared resonance wavelength coincides with the wavelength of the source laser we used in this work (~800 nm), which can induce enhanced plasmonic resonance to intensely heat up the NPs to excite the supercavitating nanobubbles. These choices also have the practical concerns that laser at this wavelength can harmlessly penetrate human tissues and conduct cell‐level light therapy [84], while most of the solid Au NPs have much shorter resonance wavelengths. The experiments are conducted in air equilibrium conditions. Degassing level might influence the nanobubble growth dynamics and size [50, 66], but as long as the supercavitation separates the NP from the surrounding liquid, these nanobubble characteristics will not influence the mechanism of the photo‐ejection of NPs.

### 4.3 MD Simulations

To use a molecular model system to test the hypothesis that supercavitation can facilitate NPs moving out of liquid, we performed MD simulations for an Au NP immersed in liquid argon using LAMMPS (Large‐scale Atomic/Molecular Massively Parallel Simulator) [85]. Two contrasting cases respectively for a nonheated (90 K) NP and an intensely heated (1000 K) NP are simulated. The temperature profiles of the Ar and the surrounding liquid as well as the density profiles for *T* = 90 K and 1000 K are shown in Figure S7 in the Supporting Information (SI5). Given the negligible impact of gravity or buoyancy in contrast to the interfacial trapping force and interatomic forces (refer to the Supporting Information, SI1), these factors have been excluded from consideration in the MD simulations. Firstly, the system consisting of an Au NP immersed in liquid argon is created, energy‐minimized, and equilibrated in a canonical ensemble (NVT) at 90 K for 5 ns with periodic boundary conditions applied in all directions. Then, the simulation box size is increased in the z‐direction to create a free space for the vapor phase. The whole structure is optimized in an isothermal–isobaric ensemble (NPT) at 1 bar and 90 K for another 5 ns to reach thermal equilibrium for the liquid‐vapor two‐phase system. For the nonheated case, the NP is given an initial velocity, so it moves toward the liquid interface, and its movement is monitored. Here we assign a z‐directed launch speed of 0–70 nm/ns (50 random trials) for the NP to reach the surface. We note that these speeds are higher than the NP in the experiments (up to 0.34 nm/ns). However, as shown, the trapping force at the surface can still prevent these high‐speed NPs from penetrating the surface [61]. For the intensely heated case, after the system is fully relaxed at 90 K, we heat the NP to 1000 K via velocity rescaling with its center of mass fixed to achieve supercavitation. Then, the NP is given an initial velocity toward the liquid surface. We then monitor the resulting dynamical behavior to study whether the NP can move across the air/liquid interface. In all simulations, a solid slab at the bottom of the simulation cell is included and maintained at 90 K using a Langevin thermostat to dissipate heat so that the overall temperature of the whole system does not rise continuously. Although 90 K is above bulk argon’s melting temperature (~84 K), the bottom slab remains crystalline since it is an artificial heat‐sink layer whose atoms interact through a much deeper cohesive potential and are lightly tethered to fixed lattice sites. For the liquid, the Lennard‐Jones argon model [86, 87], $E_{L}(r)=4\text{ε}\left[{\left(\frac{\sigma }{r}\right)}^{12}-{\left(\frac{\sigma }{r}\right)}^{6}\right]$, is used with σ=3.405 Å and $\text{ε}=0.24\;\frac{\text{kcal}}{\mathrm{mol}}$. For the NP, which has a radius of 1 nm, the Morse potential for Au [88] is used: $E_{NP}(r)=D_{0}[e^{-2\text{α}(r-r_{0})}-2e^{-\text{α}(r-r_{0})}]$, where $D_{0}=10.954\;\frac{\text{kcal}}{\mathrm{mol}}$ is the bond‐dissociation energy, $r_{0}=3.042\;Å$ is the equilibrium bond length, and α=1.583 Å is the parameter characteristic of the atom. The NP interacts with the liquid atoms also via the L‐J potential with parameters ${\text{σ}}_{\mathrm{NP}-L}=3.405\;Å$ and ${\text{ε}}_{\mathrm{NP}-L}=0.46\;\frac{\text{kcal}}{\mathrm{mol}}$ to model the hydrophilic surface. All interactions are truncated at $r_{c}=12.5\;Å$. As a comparative case to study the effect of interfacial interaction strength, we increased the energy constant of the L‐J potential by 10 times and performed the same simulation. It is worth mentioning that the distance between the NP with the slab is much greater than the force cutoff distance, so there is no influence from the slab on the dynamics of the NP. A time step of 5 fs is used for all MD simulations.

To further validate our findings, we conducted a more realistic simulation using LAMMPS, where the water molecules were modeled by the SPC/E model [89], which is known for providing accurate dynamics and structure for bulk water [90]. The non‐bond interactions between Au atoms and water molecules were simulated using the L‐J interaction, as previously described. For the Au NP, we employed the Morse potential with a force cutoff threshold of 8 Å, while the L‐J interactions were applied within a cutoff of 10 Å. To account for the long‐range electrostatic interactions within the system, we employed the Particle–Particle Particle–Mesh (PPPM) method [91], characterized by an accuracy of 10^−5^. During these simulations, we adopted a timestep of 1 femtosecond. The simulation process began with energy minimization and equilibration in an NVT ensemble at 300 K for 2 ns. Subsequently, the system underwent optimization in an NPT ensemble at 1 atm and 300 K for an additional 3 ns. Once the structures were fully relaxed, the NP (diameter = 1 nm) was heated up to 1000 K to observe the photo‐ejection phenomenon. Figure S8 in the Supporting Information (SI5) and the Supporting Movies M9 and M10 illustrate the NP, embedded in the realistic water simulation, crosses the liquid/gas interface. This outcome aligns with the previously established observations, further consolidating our proposed mechanism regarding this phenomenon. To ensure the robustness of our results and to eliminate model dependence, we also performed simulations using the TIP3P water model (Figure S9) [92, 93, 94], confirming the observation of the photo‐ejection phenomenon. If the NPs are right at the liquid–air interface, then localized heating may indeed expel the NPs out of the liquid, without the need of optical forces to move them to the interface. However, since the NPs are stably suspended in the liquid, it means their surfaces are hydrophilic. Therefore, the NPs should not be floating at the liquid‐air interface. By employing these comprehensive simulation approaches, we strengthen the validity and significance of our simulation findings.

### 4.4 Deposition of Photo‐Ejected Au NPs from Liquid

A glass substrate is used to collect the Au NPs ejected from liquid under the droplet (Figure 1a). The distance between the bottom substrate and droplet is ~ 0.5 mm in point deposition (Figure 3c–e) and 200 μm in the line deposition (Figure 4b). Each deposition process lasts for 2 min continuously. During line deposition, the bottom substrate is moved by a translation stage. To image the deposited Au NPs, a dark‐field optical microscope (Olympus B‐51) and a back‐scattered SEM (Magelllan 400, FEI Company), with working voltage 10 kV, working current 0.2 ~ 0.4 nA, and working distance 4.3 mm, are used. The sample is coated with a 2‐nm Ir layer before SEM imaging and energy‐dispersive X‐ray (EDX) spectrum measurements.

### 4.5 2D Material Nanotent Fabrications and AFM Characterization

The WSe_2_ crystals were synthesized using a self‐flux method, as described in the literature (Supporting Information, SI11) [95]. Monolayer WSe_2_ was subsequently exfoliated onto a SiO_2_/Si substrate through gold‐assisted exfoliation [96]. To confirm the monolayer nature of the exfoliated WSe_2_, photoluminescence spectroscopy was employed (Supporting Information, SI11). This was achieved using a Horiba LabRam HR evolution system equipped with a 532‐nm laser and a 100× objective (NA = 0.9). The laser power was set at approximately 25 µW, and the acquisition time was 0.5 s. For the transfer of the WSe_2_ monolayer onto an Au NP/quartz substrate (Figure 5d), a wet pick‐up and dry transferring method was employed [97]. To elaborate, a PDMS stamp (Gel‐Pack Gel‐Film X4) was brought into contact with the monolayer WSe_2_ on the SiO_2_/Si substrate. Subsequently, a mixture of water and 2‐propanol in a ratio of approximately 5:1 was injected onto the sample. This facilitated the delamination of the WSe_2_ from the substrate, allowing it to adhere to the PDMS. Following this, the WSe_2_ on the PDMS stamp was dried using nitrogen. Finally, the WSe_2_ was brought into contact with the Au NPs on the quartz substrate, and the PDMS stamp was removed. To conduct the transferring AFM tapping mode topography measurement, a commercially available AFM (Asylum Research, Cypher ES) was used in ambient conditions. A standard tapping mode AFM probe, specifically the AC 160 (MikroMasch), was employed for this purpose.

## Supporting Information

Additional supporting information can be found online in the Supporting Information Section. **Supporting Fig. S1:** The model (a) and calculated profile (b) of the trapping force of an NP (blue: r = 60 nm, orange: r = 1 nm) while it penetrates the curved water/air interface. **Supporting Fig. S2** **:** The normalized field profiles of complex electric field amplitude (a) and the z‐component of time‐averaged Maxwell’s stress tensor (b) profiles of an NP (radius: 60 nm) with a nanobubble (radius: 130 nm). **Supporting Fig. S3** **:** Temperature profile of a NP in water after a single 800 nm laser pulse (7 mJ cm^−2^). The particle snaps from room temperature to ~1680 K in <1 ps and then cools to ~1310 K over 15 ps—well above water’s spinodal threshold, ensuring vapor‐sheath formation. **Supporting Fig. S4:** The geometry and the boundary conditions of the simulated system. **Supporting Fig. S5** **:** (a) Schematic of the pump‐probe optical scattering imaging experiment. Dark‐field optical scattering images (b) without pump laser and (c) with pump laser. The green spots correspond to the diffraction limited scattered probe light from the Au NPs with nanobubbles. **Supporting Fig. S6** **:** MD simulation snapshots of an intensely heated NP (T = 1000 K) with supercavitation moving out of liquid after we increased the energy constant of the L‐J potential by 10 times. **Supporting Fig. S7:** The local liquid (a) temperature and (b) density distribution in the close vicinity of the NP for TNP = 90 K and 1000 K. The insets in plot (a) and plot(b) show the sliced view of the TNP = 90 K and 1000 K, respectively. Distance denotes the radial distance from the NP center. Temperature and density were computed in 2 Å spherical shells and angle averaged. **Supporting Fig. S8** **:** MD simulation snapshots of (a) an intensely heated NP (T = 1000 K) with supercavitation moving out of the liquid and (b) an NP (T = 300 K) at room temperature without supercavitation trapped at the liquid interface with the realistic water models (SPC/E). **Supporting Fig. S9** **:** MD simulation snapshots of (a) an intensely heated NP (T = 1000 K) with supercavitation moving out of the liquid and (b) an NP (T = 300 K) at room temperature without supercavitation trapped at the liquid interface with the realistic water models (TIP3P). **Supporting Fig. S10** **:** (a) The model, boundary conditions and mesh structures used in the simulations of the temperature profiles in the suspension droplet upon laser irradiation. (b) The simulated 2D temperature profile on the plane crossing the center axis of the droplet for case III in the main text (**Figure 3b**). **Supporting Fig. S11** **:** The temperature increase from room temperature of the droplet along the arc line with (black) or without (red) the Marangoni effect. **Supporting Fig. S12:** Dark‐field optical images of the Au NPs photo‐ejected from liquid and deposited on the bottom glass substrate in (a) point deposition and (b) line deposition experiments with **Supporting Fig. S13:** The MD‐simulated spread angles of 18 different NPs. **Supporting Fig. S14:** The bright‐field optical image (a) and photoluminescence spectroscopy (b) of the fabricated monolayer WSe_2_. **Supporting** **Table** **S1:** The Morse potential parameters for interactions within Au and water. **Supporting Movie M1:** Dark‐field laser probing of the photo‐ejected Au NPs across the air/liquid interface. **Supporting Movie M2:** Dark‐field transient optical scattering imaging with only the probe laser. **Supporting Movie M3:** Dark‐field transient optical scattering imaging with both the pump and probe lasers. **Supporting Movie M4:** MD simulated microscopic mechanism of the NP stranded at the liquid/air interface due to the capillary force. **Supporting Movie M5:** MD simulated microscopic mechanism of the supercavitating NP moving out of liquid interface. **Supporting Movie M6:** Dark‐field optical probing of the stochastic nature of the supercavitating NP motions with the laser focal point inside liquid suspension (without probe laser). **Supporting Movie M7:** MD simulated supercavitating NP moving out of liquid interface with a large spread angle. **Supporting Movie M8:** MD simulated supercavitating NP moving out of liquid interface with a small spread angle. **Supporting Movie M9:** MD simulated microscopic mechanism of the NP stranded at the liquid/air interface due to the capillary force in the realistic water model. **Supporting Movie M10:** MD simulated microscopic mechanism of the supercavitating NP moving out of liquid interface in the realistic water model.

## Author Contributions

Q.Z., S.M., M.R.R., E.L., and T.L. designed the experiments. Q.Z. and Y.Y. set up and performed the experiments. D.H., Q.Z., R.Z., A.M., E.L., and T.L. designed the simulations. R.Z. and D.H. performed the simulations. D.H., R.Z., A.M., and Q.Z. wrote the manuscript, and J.C.H., J.S., M.R.R., E.L., and T.L. revised it.

## Funding

This study was supported by National Science Foundation (Grant 1706039), Center for the Advancement of Science in Space (Grant GA‐2018‐268), and National Research Foundation of Korea (NRF) grant funded by the Korea government (MSIT)(RS‐2025‐16064483).

## Conflicts of Interest

The authors declare no conflicts of interest.

## Supporting information

Supplementary Material

## Data Availability

The data that support the findings of this study are included in the main text and Supporting Information, as well as the 10 Supporting Movies.
