# Supplementary material for: Plasmonic Supercavitation Enables Nanoparticle Photo‐Ejection Across Air/Water Interface
Source: Small Sci. 2026 Mar 23;6(3):e202500563. doi: 10.1002/smsc.202500563 (PMC13097346; doi:10.1002/smsc.202500563)
Supplement: Supplementary file 1 — Supplementary Material [file SMSC-6-e202500563-s001.pdf]

Supporting Information for

Plasmonic Supercavitation Enables Nanoparticle Photo-Ejection across Air/Water Interface

Qiushi Zhang[1], Renzheng Zhang[1], Amartya Mandal[1], Dezhao Huang[1,5], Yucheng Yang[1], Seunghyun Moon[1], Jarrod Schiffbauer[2], Daniel A. Rhodes[6], James C. Hone[7], Matthew R. Rosenberger[1], Eungkyu Lee[3,*], and Tengfei Luo[1,4,*]

1. Department of Aerospace and Mechanical Engineering, University of Notre Dame, IN, USA
2. Department of Physical and Environmental Sciences, Colorado Mesa University, Co, USA
3. Department of Electronic Engineering, Kyung Hee University, Yongin-si, South Korea
4. Department of Chemical and Biomolecular Engineering, University of Notre Dame, IN, USA
5. School of Power and Mechanical Engineering, Wuhan University, Hubei, China
6. Department of Materials Science and Engineering, University of Wisconsin-Madison, WI, USA
7. Department of Mechanical Engineering, Columbia University, NY, USA

* Corresponding authors: eleest@khu.ac.kr; tluo@nd.edu.

Legends for Supporting Movies:

***Supporting Movie M1*:** Dark-field laser probing of the photo-ejected Au NPs across the air/liquid interface.

***Supporting Movie M2*:** Dark-field transient optical scattering imaging with only the probe laser.

***Supporting Movie M3*:** Dark-field transient optical scattering imaging with both the pump and probe lasers.

***Supporting Movie M4*:** MD simulated microscopic mechanism of the NP stranded at the liquid/air interface due to the capillary force.

***Supporting Movie M5*:** MD simulated microscopic mechanism of the supercavitating NP moving out of liquid interface.

***Supporting Movie M6*:** Dark-field optical probing of the stochastic nature of the supercavitating NP motions with the laser focal point inside liquid suspension (without probe laser).

***Supporting Movie M7*:** MD simulated supercavitating NP moving out of liquid interface with a large spread angle.

***Supporting Movie M8*:** MD simulated supercavitating NP moving out of liquid interface with a small spread angle.

***Supporting Movie M9*:** MD simulated microscopic mechanism of the NP stranded at the liquid/air interface due to the capillary force in the realistic water model.

***Supporting Movie M10*:** MD simulated microscopic mechanism of the supercavitating NP moving out of liquid interface in the realistic water model.

SI1. Estimation of the interfacial trapping force on a NP at the curved water/air interface

The trapping force of a small particle at the interface depends on its size and contact angle (location relative to the interface) of it.[1–3] To calculate the trapping force of a NP at the curved water/air interface (see **Figure 2a** in the main text for schematic), we first need to calculate the trapping force at a flat interface:[4,5]

$$F_{flat} = -\frac{\pi r^2 \gamma (1-|\cos\theta|)^2}{\Delta z} \qquad \text{(s1)}$$

where r is the radius of the NP (60 nm), γ is the surface tension of water ($\sim 0.07\frac{N}{m}$), θ is the contact angle and Δz is the displacement of the NP in the air with respect to the water/air interface ($\sim 100\ nm$).[6,7] However, due to the curved profile of the NP suspension droplet that we used in this work (**Figure 1a**), we have to include a correction term in the force calculation:[4]

$$F_{correction} = \frac{\pi \gamma a^4}{\Delta z}\left(\frac{3}{16}H^2\right) \qquad \text{(s2)}$$

where a is the distance from the contact line to the central axis of the NP, and H is the curvature of the semispherical droplet ($H = 2/R$, where R is the radius of the droplet, 1 mm). Thereby, the total trapping force of an NP at the curved interface is as follows:

$$F_{curved} = |F_{flat} - F_{correction}| \qquad \text{(s3)}$$

While the NP penetrates through the droplet interface (**Figure S1a**), the instantaneous θ (0 ~ 90 ~ 180 degrees) and a (0 ~ r ~ 0) will vary accordingly, giving us the symmetric trapping force profile as shown in **Figure S1b**. Therefore, we can estimate the magnitude of the trapping force on a 120 nm NP at the curved interface to be $\sim 10^{-8}$ N. For a 2 nm NP, the trapping force at the curved interface is estimated to be $\sim 10^{-10}$ N.

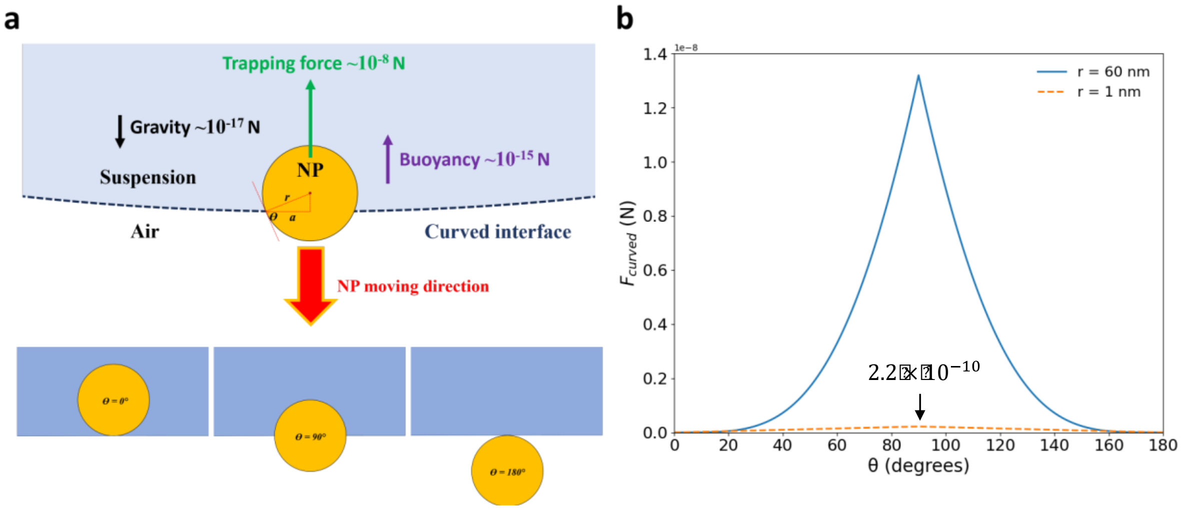

Figure S1. The model (a) and calculated profile (b) of the trapping force of an NP (blue: r = 60 nm, orange: r = 1 nm) while it penetrates the curved water/air interface.

To note, for a gold/silica core-shell NP that used in this work, with a radius of 60 nm, the magnitude of gravitational force (along the NP moving direction) is only ~8×10^{-17} N. In addition, as we discussed in section SI3, the supercavitating nanobubble usually has a radius of a few hundred nm. For a nanobubble with a diameter of 1 μm, the buoyancy force is ~5×10^{-15} N. Therefore, the net force from gravitational and buoyancy in the NP moving axis should be in the order of 10^{-15} N, which is negligible compared to either the trapping force (~10^{-8} N, section SI1) or the optical scattering driving force (~10^{-12} N, section SI2).

SI2. Calculation of the dispersive optical scattering force on an Au NP

As discussed in ref. [8], the dispersive optical scattering force on an NP by a pulsed laser is a function of time, which can be calculated by the Lorentz's force density (**f**) equation on a small object:

$$\mathbf{f} - \frac{\partial \mathbf{S}}{\partial t} = \nabla \cdot T \tag{s4}$$

where T is the Maxwell's stress tensor and **S** is the electromagnetic momentum density.[9–11] While the incident light is a function of time, the Lorentz's force density (**f**) is also a function of time, t.

However, the oscillation of the amplitude of electromagnetic field at the optical frequency is convoluted by the duration of a pulse for our pulsed incident light with the duration of ~94 fs and the optical frequency of 3.7×10^{14} Hz (= a period of ~2.7 fs). It is obvious that these time scales are much faster than the mechanical response of a supercavitating NP, which suggests that the time-averaged optical force should be appropriate for analyzing the motion of NPs.[12–14] By using equation (s4), the time-averaged force $\mathbf{F}_t$ on an NP can be written as:

$$\mathbf{F}_t = \frac{1}{t_2 - t_1}\{\int_{t_1}^{t_2} \oint T \cdot \mathrm{d}A\mathrm{d}t - \int \mathbf{S}_{t_2}\mathrm{d}V + \int \mathbf{S}_{t_1}\mathrm{d}V\} \tag{s5}$$

where A and V are the surface and volume of an NP, respectively.

Because the pulsed laser has a repetition rate of ν_0 = 80.7 MHz, the last two volume integral terms in equation (s5) will vanish, if we pick t_1 and t_2 to integrate over one pulse, as they are identical. It means that the Maxwell's stress tensor alone is sufficient to determine $\mathbf{F}_t$ without any momentum density terms from the perspective of the time-averaged force calculation. Besides, as discussed in ref. [8], we can use the optical force from a continuous

wave laser with the same central frequency and intensity to approximate the time-averaged force on an Au NP by the a pulsed laser. Therefore, the time-averaged optical force on an Au NP ($\mathbf{F}_t$) can be estimated by the equation below:

$$\mathbf{F}_t \cong \oiint T_t \cdot \boldsymbol{n} \ \mathrm{da} \tag{s6}$$

where T_t is the time-averaged Maxwell's stress tensor,[15,16] and $\boldsymbol{n}$ is the normal vector of the surface of the Au NP. The time-averaged Maxwell's stress tensor is formulated with the electromagnetic field profiles of the simulation domain, which can be obtained by solving the Maxwell's equations at a frequency domain (i.e., at the central wavelength, 800 nm) with finite element method (*i.e.*, *COMSOL Multiphysics*). The normalized field profiles of complex electric field amplitude and the z-component of T_t of an NP (radius: 60 nm) with a nanobubble (radius: 130 nm) system are shown in **Figure S2**. By integrating the time-averaged Maxwell's stress tensor over the whole surface of NP, we can estimate $\mathbf{F}_t$. The z-component of $\mathbf{F}_t$ heads to the laser propagation direction. Note, the force on a bare NP is slightly larger than but of the same order of magnitude as a supercavitating NP. In addition, the optical force on the NP does not change order of magnitude when the nanobubble size changes.[8]

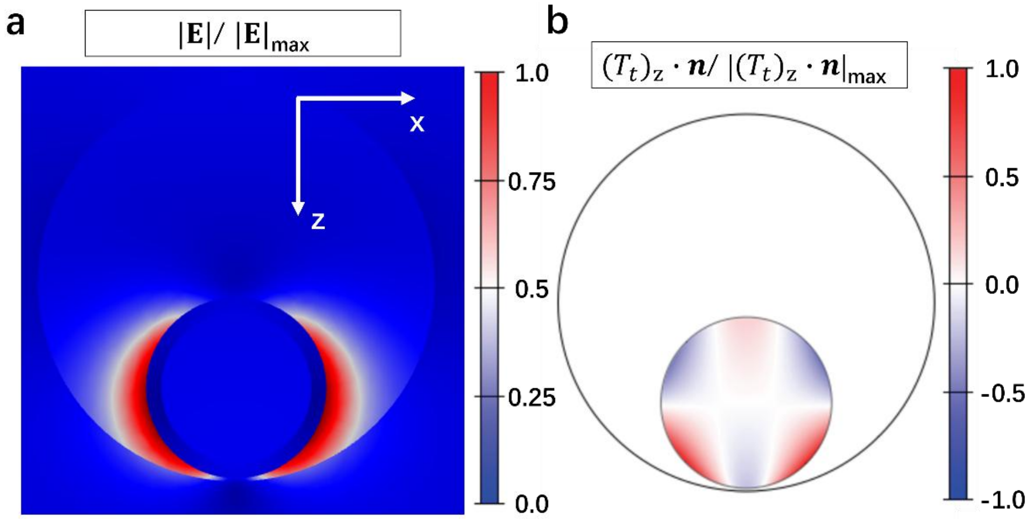

Figure S2. The normalized field profiles of complex electric field amplitude (a) and the z-component of time-averaged Maxwell's stress tensor (b) profiles of an NP (radius: 60 nm) with a nanobubble (radius: 130 nm).

SI3. Temperature increase corresponding to the supercavitation threshold of 7 mJ/cm2

Using Mie-theory calculations for a 100 nm SiO_2 core with a 10 nm Au shell, the absorption cross-section at 800 nm is σ abs = 5.86×10^3 nm² (5.86×10^{-15} m²). A single pulse at the experimentally determined threshold (7 mJ cm⁻²) therefore deposits E $\approx 4.1 \times 10^{-13}$ J into one NP. The result, plotted in **Figure S3**, shows the lattice temperature climbing from 293.15 K to a peak of ≈ 1680 K within 0.7 ps and relaxing to ≈ 1310 K after 15 ps. The numerical threshold fluence, the calculated σ_{abs}, and the simulated temperature rise are all internally consistent with the onset of nanobubble formation that underpins the photo-ejection mechanism. To assess inter-pulse heating at 80 MHz, we extended the temperature decay to the ns regime using a diffusion-limited cooling time constant $\tau \sim \frac{r^2}{\alpha}$ for a ~120-nm Au NP in water (τ = 26 ns). This yields T(12.5 ns) ≈ 920 K, indicating that the NP temperature remains high if we do not consider evaporation bringing away heat. However, the evaporation of water during the bubble formation process will cool down the NP, prevents excessive temperature rise or shell damage.

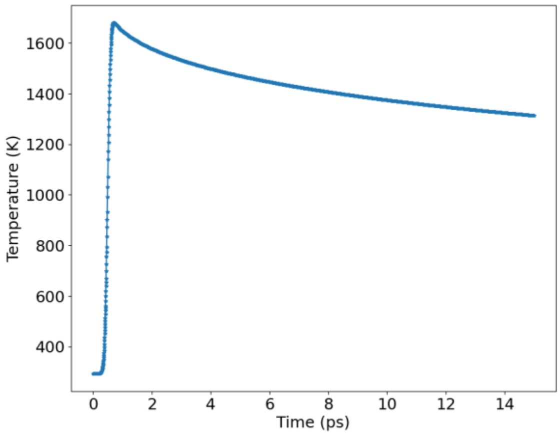

Figure S3. Temperature profile of a NP in water after a single 800 nm laser pulse (7 mJ cm⁻²). The particle snaps from room temperature to ~1680 K in <1 ps and then cools to ~1310 K over 15 ps—well above water's spinodal threshold, ensuring vapor-sheath formation.

The simulation is performed using COMSOL Multiphysics. We used an axis symmetric

component to define the system as shown in **Figure S4**. The system consists of the water domain (200 um × 200 um), and the nanoparticle (diameter = 120 nm) is centered in the water domain.

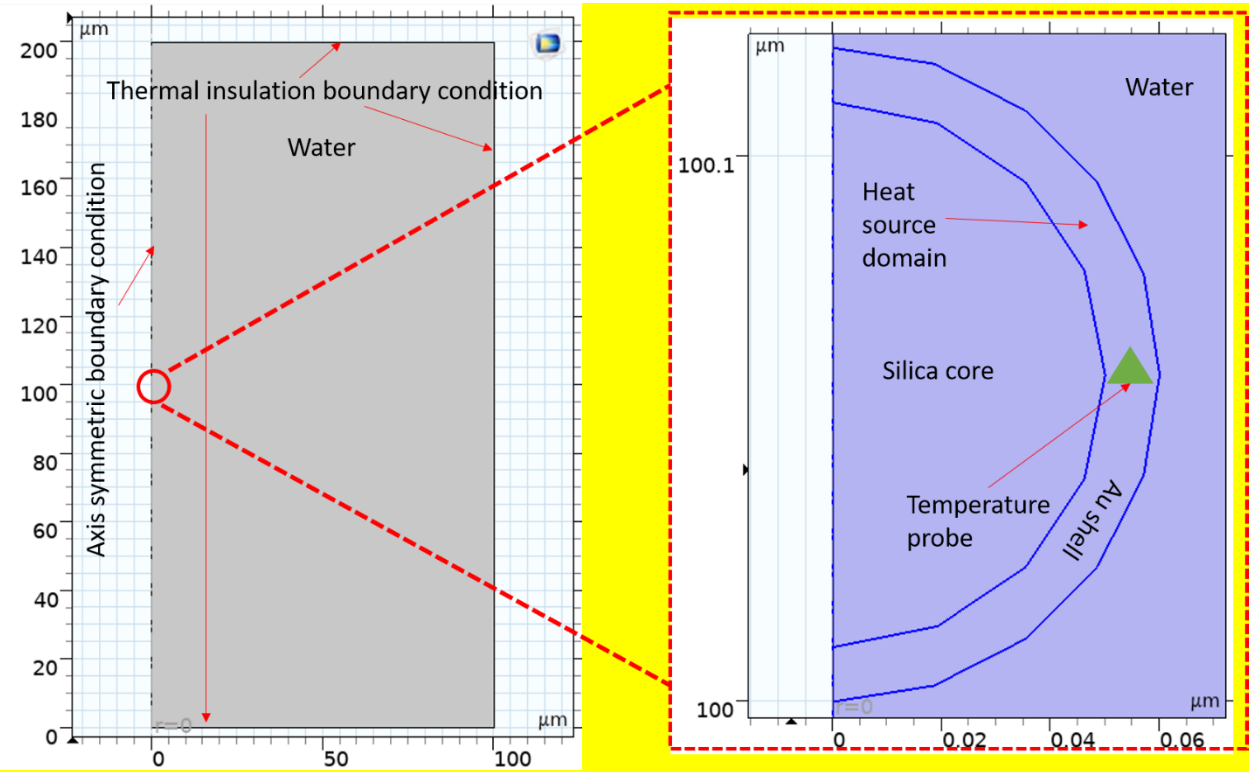

Figure S4. The geometry and the boundary conditions of the simulated system.

We used a time-dependent heat transfer model in COMSOL and set the Au shell as the heat source, as it is the material that absorbs the light energy. To define the light pulse, we utilized discrete and explicit events to generate periodic pulses with 200 fs width with 12.5 ps periodicity. The heat source is then defined as follows [17,18]:

$$Q = \frac{F\sigma_{abs}}{V} event(t) \qquad (s7)$$

Here F is the fluence, σ_{abs} is the absorption cross-section of the NP, and V is the volume of the Au shell. The pulse event as mentioned before is a function of time (t). We used axial symmetry

at the axis, and thermal insulation on the outermost boundary of the water domain. Finally, the temperature is probed at the middle of the Au shell as shown in **Figure S4**.

We note that the heat pulse on the NP in water can lead to a temperature of 920K. This correspond to the scenario of the first pulse, but the results show that the temperature is sufficiently high for vapor bubble generation.

SI4. Pump-probe optical scattering imaging experiment confirming the formation of supercavitating nanobubbles

To confirm the formation of nanobubbles around plasmonic NPs, we employed the pump-probe optical scattering imaging technique (see **Figure S5a** for the experimental setup).[8,19,20] This method uses a pump light with specific characteristics, including a femtosecond laser pulse, a wavelength of 800 nm, a repetition rate of 80.7 Mhz, and a power of ~690 mW, along with a probe light that is a continuous wave laser with a wavelength of 533 nm and a power of ~5 mW. The pump light has a wavelength that coincides with the surface plasmon resonance (SPR) of the coreshell Au NPs in water used in the NP photo ejection experiment, while the probe light has a wavelength that is away from the SPR of the Au NPs. Both lights are introduced as Gaussian beams to pass through an objective lens (20×) and into the Au NP suspension contained in a quartz cuvette, as illustrated in **Figure S5a**.

For detection, another objective lens (10×) is focused on the cross-plane of the NP suspension volume where the optical axes of the pump beam and the probe beam overlap. The scattered light from the cross-plane sequentially passes through the 10× objective lens, a mechanical pinhole, a low-frequency bandpass filter, a tube lens, and then is focused onto the image sensor of a high-speed camera with ultra-high light sensitivity (HX-7, color ISO rating of 8000, 10,000 FPS). The mechanical pinhole enhances sensitivity by allowing the scattered

light at a certain solid angle to pass through, while the low-frequency bandpass filter blocks any scattered pump light and only permits scattered probe light to pass through. Using the experimental technique illustrated in **Figure S5a**, we can detect changes in the scattered probe light intensity at a specific solid angle on the cross-plane when the pump beam is applied to Au NPs in the suspension. Our observations show that without the pump beam, the camera fails to detect any noticeable scattered probe light (**Figure S5b**, also see Supporting Movie M2). However, when the pump beam is used, diffraction-limited green spots appear on the cross-plane, indicating a significant modification of the far-field spatial intensity profile of the scattered probe light from Au NPs (**Figure S5c**, also see Supporting Movie M3). Such modification of scattered light can only occur when the Au NP is coupled to object(s) located within the subwavelength length scale. Based on our findings, we interpret the objects responsible for the modification as nanobubbles on Au NPs, consistent with previous research using similar optical scattering imaging techniques.[19,21,22]

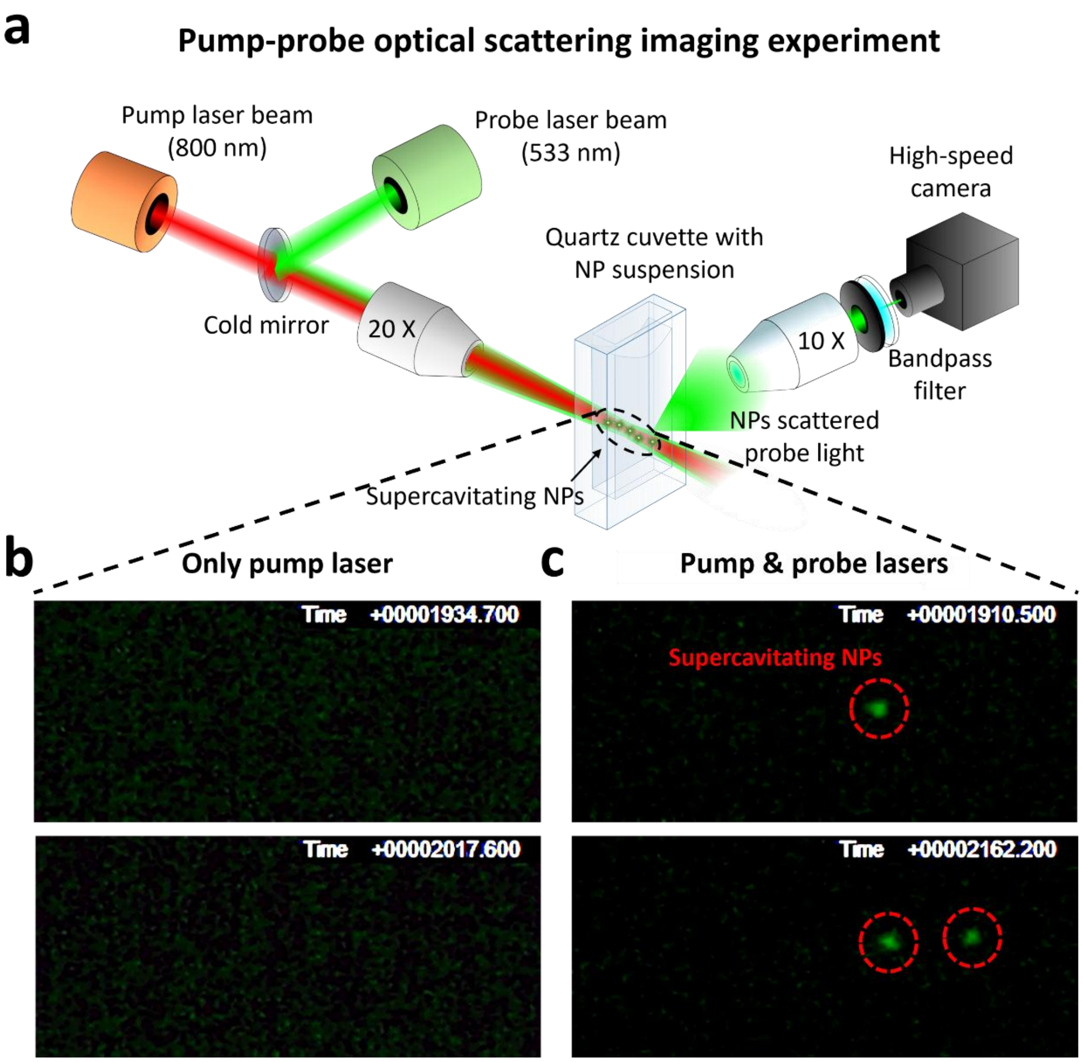

Figure S5. (a) Schematic of the pump-probe optical scattering imaging experiment. Dark-field optical scattering images (b) without pump laser and (c) with pump laser. The green spots correspond to the diffraction limited scattered probe light from the Au NPs with nanobubbles.

SI5. MD simulations showing the supercavitating NP ejection with stronger and realistic NP-liquid interfacial interaction

For the water simulation, we use the SPC/E (extended) water model. Parameters are listed below:

O_{mass} = 15.9994, H_{mass} = 1.008, O_{charge} = -0.8476, H_{charge} = 0.4238, LJ energy constant of OO = 0.1553, LJ distance constant of OO = 3.166, LJ energy and distance constant of OH, HH = 0.0, length of OH bond r_0 = 1.0 Å, and the HOH angle θ_0 = 109.47°

For the gold simulation, we use the Morse potential:

$$U(r) = D_0[(1 - e^{-\alpha(r-r_0)})^2 - 1]$$

where D_0 is the bond-dissociation energy, r_0 is the equilibrium bond length, and α is Range/steepness parameter. Larger α makes the well narrower and the repulsive wall steeper.

Table S1 summarizes the Morse potential parameters for interactions within Au and water. "Ho" and "Ow" represents the hydrogen atom and the oxygen atom in water molecules.

Table S1. The Morse potential parameters for interactions within Au and water

**Pair type**	**D_0, kcal/mol**	**α, 1/Å**	**r_0, Å**
Au-Au	10.954	1.583	3.042
**Pair type**	**ϵ, kcal/mol**	**σ, Å**	
Au-Ho	0	0	
Au-Ow	0.141	3.6	

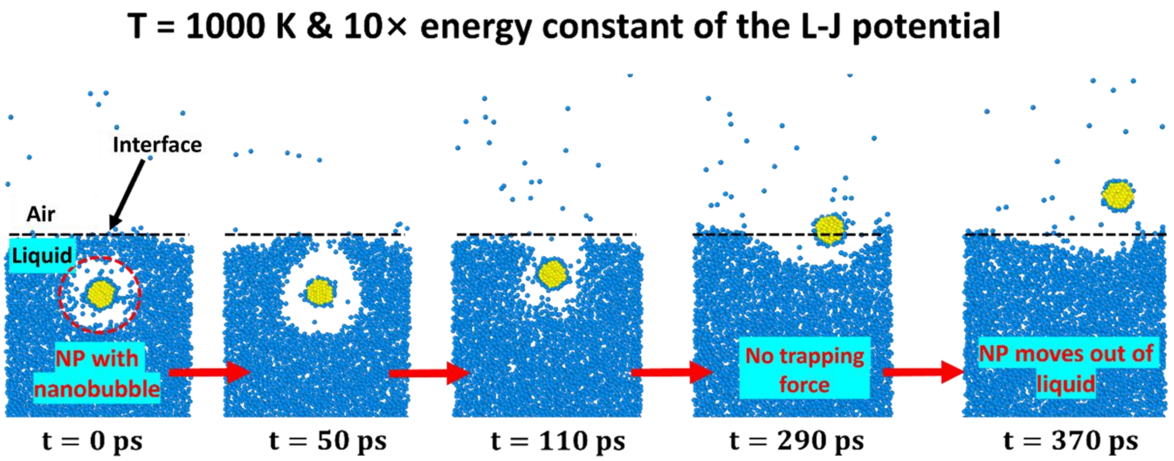

Figure S6. MD simulation snapshots of an intensely heated NP (T = 1000 K) with supercavitation moving out of liquid after we increased the energy constant of the L-J potential by 10 times.

After minimization, systems were equilibrated for 2 ns (NVT, 300 K) and 3 ns (NPT, 1 atm, 300 K). Production runs were carried out in the NVE ensemble for 1 ns with the NP set either to 90 K or 1000 K for comparison. Temperature profiles were computed from frames collected every 10 ps and averaged over the last 1 ns of the production run.

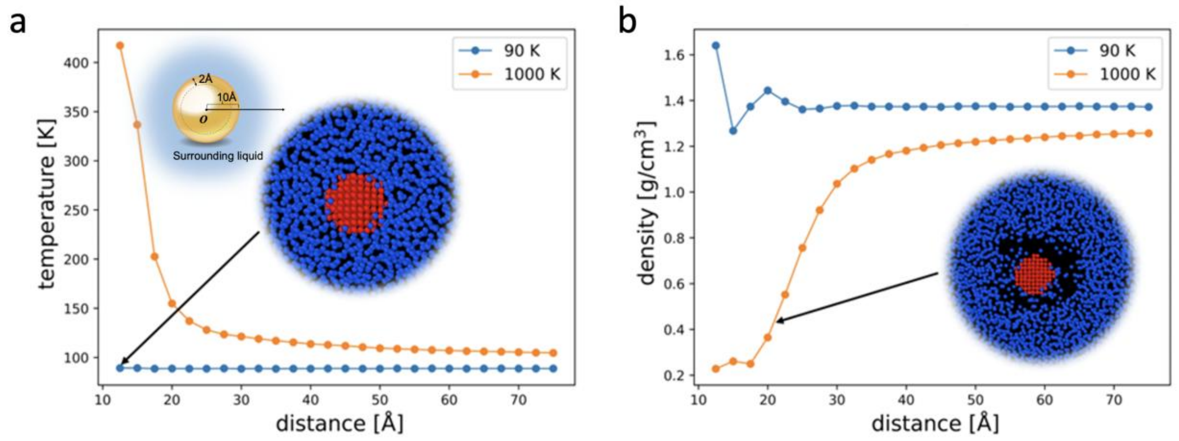

Figure S7. The local liquid (a) temperature and (b) density distribution in the close vicinity of the NP for TNP = 90 K and 1000 K. The insets in plot (a) and plot(b) show the sliced view of the TNP = 90 K and 1000 K, respectively. Distance denotes the radial distance from the NP

center. Temperature and density were computed in 2 Å spherical shells and angle averaged.

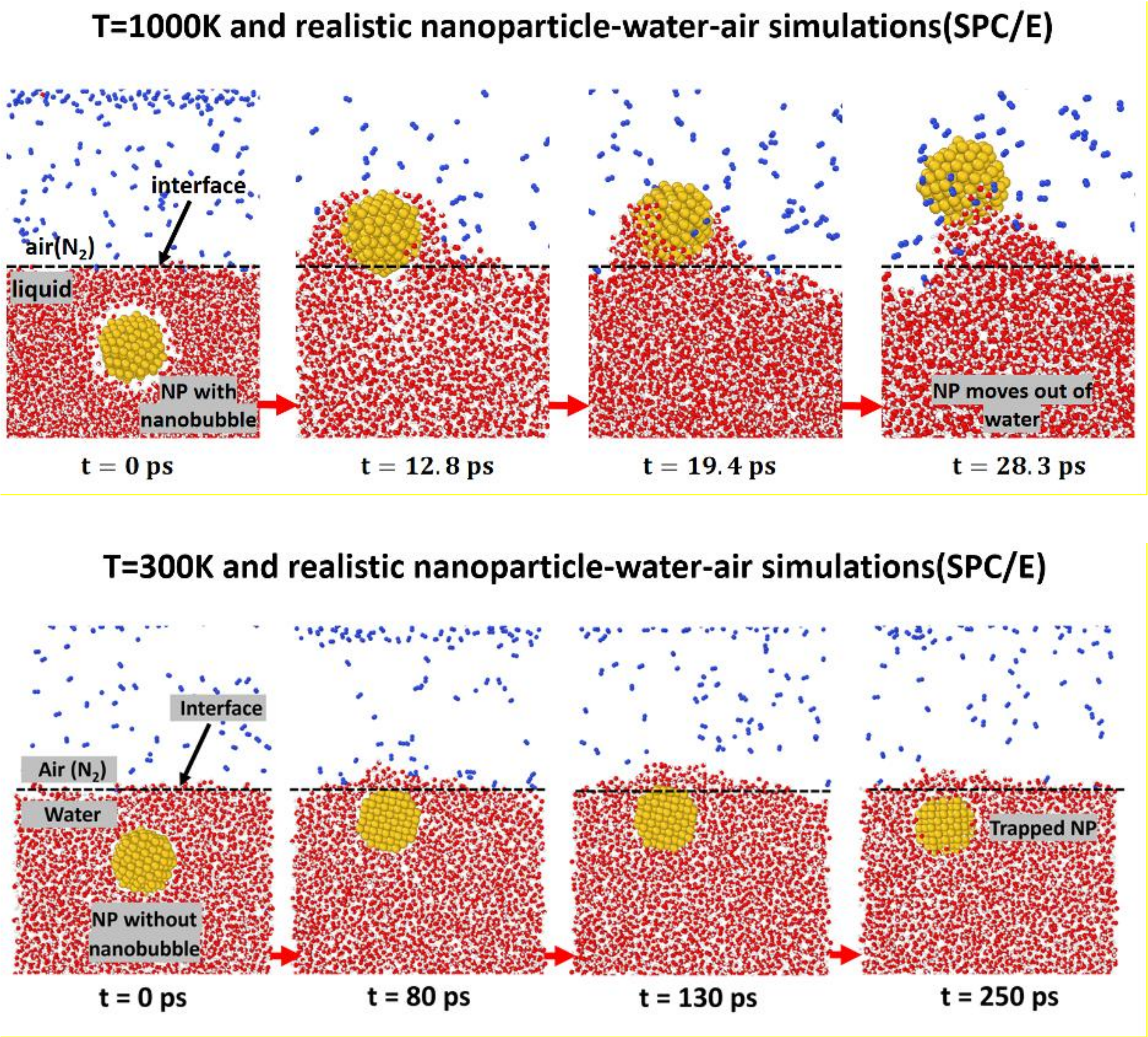

Figure S8. MD simulation snapshots of (a) an intensely heated NP (T = 1000 K) with supercavitation moving out of the liquid and (b) an NP (T = 300 K) at room temperature without supercavitation trapped at the liquid interface with the realistic water models (SPC/E).

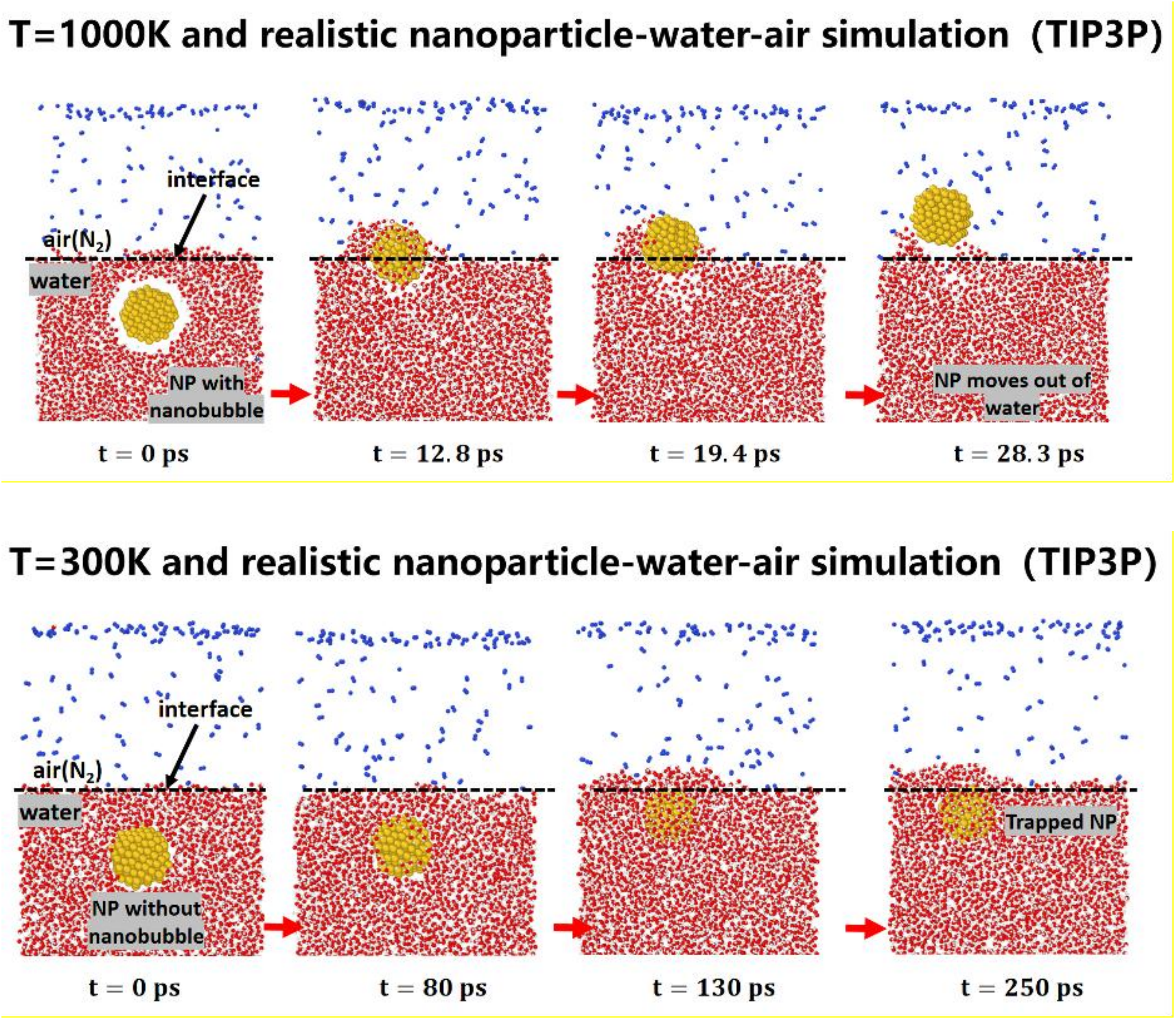

Figure S9. MD simulation snapshots of (a) an intensely heated NP (T = 1000 K) with supercavitation moving out of the liquid and (b) an NP (T = 300 K) at room temperature without supercavitation trapped at the liquid interface with the realistic water models (TIP3P).

SI6. Finite element thermo-fluidic simulations details

We used *COMSOL Multiphysics* to calculate the temperature profile of the NP suspension droplet under the irradiation of a Gaussian laser beam. The flow effect, heat conduction and the laser attenuation effect of NP suspension are included in our simulations. The model and mesh structures used in our simulations are shown in **Figure S10a**. The model includes a layer of SiO_2 substrate on the top. A water hemisphere with much finer mesh structures represents the suspension droplet. The dimension of each component is similar to that in our experiments. The incident laser beam propagates along the z-direction (**Figure S10a**). As discussed in refs. [23,24], the incident laser induces volumetric heating inside the droplet due to absorption from the suspended Au NPs, and the heat generation rate decays along the laser propagation direction by an attenuation factor of the Au NP suspension we used in our experiments.

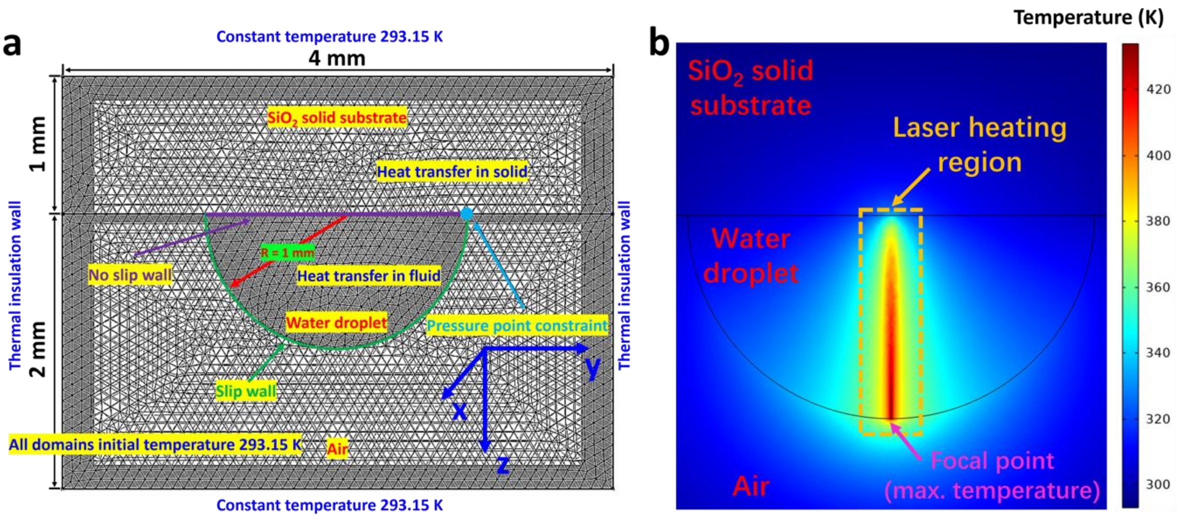

Figure S10. (a) The model, boundary conditions and mesh structures used in the simulations of the temperature profiles in the suspension droplet upon laser irradiation. (b) The simulated

2D temperature profile on the plane crossing the center axis of the droplet for case III in the main text (**Figure 3b**).

There are several conditions that have been assumed in our simulations: (1) The liquid flow and heat transfer are at steady state. (2) In the liquid water, the flow is laminar and incompressible without body forces, which satisfies the following momentum equation:

$$\rho(\vec{u}\cdot\nabla)\vec{u}-\nabla\cdot\left(\mu\left(\overleftrightarrow{\nabla u}+\overleftrightarrow{\nabla u}^{\mathrm{T}}\right)-p\overleftrightarrow{I}\right)=0 \tag{s8}$$

, and continuity equation:

$$\rho(\nabla\cdot\vec{u})=0 \tag{s9}$$

where ρ is the density of water, μ is the dynamic viscosity of water, $\vec{u}$ is the velocity vector, p is the pressure, and $\overleftrightarrow{I}$ is a 3x3 identity tensor. (3) The air medium and SiO_2 substrate are considered as non-fluidic rigid materials with conduction being the only mode of heat transfer. (4) The volumetric heating of the Au NPs irradiated by laser is the only heat source in the system, which supplies the heat to the liquid water droplet with the following heat transfer equations:

In water,

$$\rho C_p\vec{u}\cdot\nabla T-k_w\nabla^2 T=Q_v \tag{s10}$$

where C_p is the heat capacity of water at constant pressure, T is the temperature, k_w is the thermal conductivity of water, Q_v is the heat generation rate by the volumetric heating.

In air and the SiO_2 substrate,

$$-k_s \nabla T = q \quad \text{(s11)}$$

where k_s is the thermal conductivity of air (or SiO_2 substrate), and q is the heat flux coming through the air/water boundary (or air/ SiO_2 substrate boundary). The boundary conditions used in our simulations are similar to those in refs. [24,25] and labeled in **Figure S10a**. The heat generation rate of the volumetric heating is as the following [24]:

$$Q_v = \eta_{abs} \cdot \alpha \cdot e^{-\alpha z} \cdot \frac{P_L}{2\pi\sigma^2[1+(\frac{\lambda(R-z)}{4\pi n\sigma^2})^2]} \cdot exp\left[-\left(\frac{x^2+y^2}{2\sigma^2[1+(\frac{\lambda(R-z)}{4\pi n\sigma^2})^2]}\right)\right] \quad \text{(s12)}$$

where P_L is the source laser power, and η_{abs} is the optical absorption efficiency of Au NPs, which is determined by the ratio of the absorption quality factor and the extinction quality factor of the Au NP in deionized water.[25] λ is the wavelength of the source laser (~800 nm) and n is the refraction index of water (~1.33). The optical attenuation factor of the NP suspension α is extracted from the absorbance spectrum.[23] For our 20× objective lens, σ = 3 μm is the standard derivation of the Gaussian beam, and R is the radius of the droplet, which is 1 mm. As we can see in equation (s11), the heat generation rate for a given location is determined by the radius of the Gaussian distribution of laser intensity along the z-direction and in the x-y plane (see **Figure S10a** for coordinate definitions). An example of simulated temperature profile for Case III in the main text (**Figure 3b**) is shown in **Figure S10b**. This is on the plane crossing the center axis of the droplet, which is also the center axis of the laser beam. The region heated up by laser is depicted in a yellow rectangular (**Figure S10b**) with the maximum temperature located at the tip of the droplet where is the laser focal point (also see **Figure 3b** in the main text).

We also considered the Marangoni effect at the interface of the droplet (air/water slip boundary, **Figure S10a**) as:

$$\left[\mu\left(\overleftrightarrow{\nabla\vec{u}} + \overleftrightarrow{\nabla\vec{u}}^{\mathrm{T}}\right) - \left(p + \frac{2}{3}\mu(\nabla \cdot \vec{u})\right)\overleftrightarrow{I}\right]\hat{n} = \gamma\nabla_t T \tag{s13}$$

where $\hat{n}$ is the normal outward vector to the surface of the droplet, γ is the temperature derivative of the water/air surface tension, and ∇_t is the gradient of the tangent vector to the surface of the droplet. As depicted in **Figure S11**, despite some variations in the temperature profile, the maximum temperature at the laser focal point only experiences a small decrease of ~5% when considering the influence of the Marangoni effect. Based on this calculation, it is reasonable to assert that the contribution of the Marangoni effect is relatively insignificant, and consequently, the maximum temperature of case III (as shown in **Figure 3** of the main text) remains at least 40 K above the boiling point.

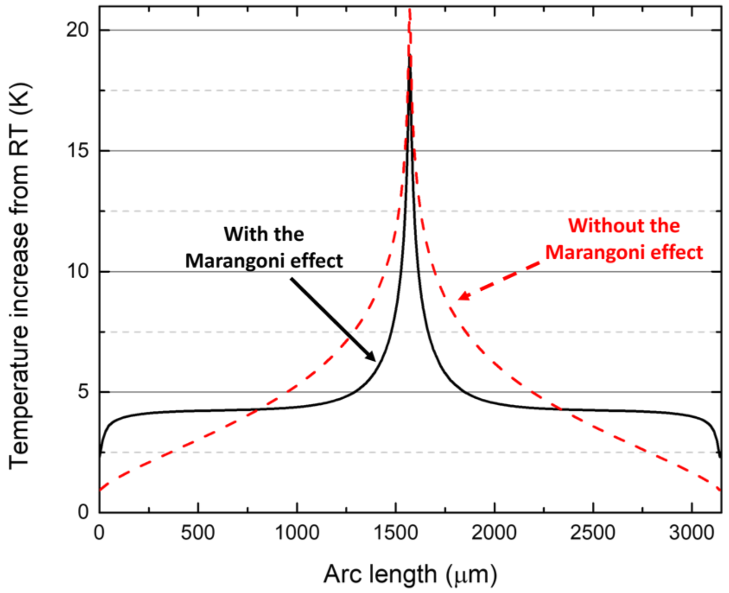

Figure S11. The temperature increase from room temperature of the droplet along the arc line with (black) or without (red) the Marangoni effect.

SI7. Water boiling point at the curved interface of a droplet

Due to the curvature of the droplet, there is a pressure difference between the inside and outside of the droplet, called Laplace pressure (ΔP). The Laplace pressure at the curved interface in our case can be calculated as follows:[4]

$$\Delta P = \gamma H \tag{s14}$$

where γ is the surface tension, $\gamma = \sim 0.07\frac{N}{m}$, and H is the curvature of the droplet ($H = 2/R$, where R is the radius of the droplet, 1 mm). The total pressure inside the droplet amounts to:

$$P_{tot} = P_{atm} + \Delta P = 101325\frac{N}{m^2} + 140\ \frac{N}{m^2} \approx 101.5\ \text{kPa} \tag{s15}$$

which corresponds to the water boiling point of 100.023 ℃. As a result, the influence of the droplet interfacial curvature on water boiling point is negligible for our millimeter-scale droplet.

SI8. Characterizing the deposited Au NPs photo-ejected from liquid onto a glass substrate

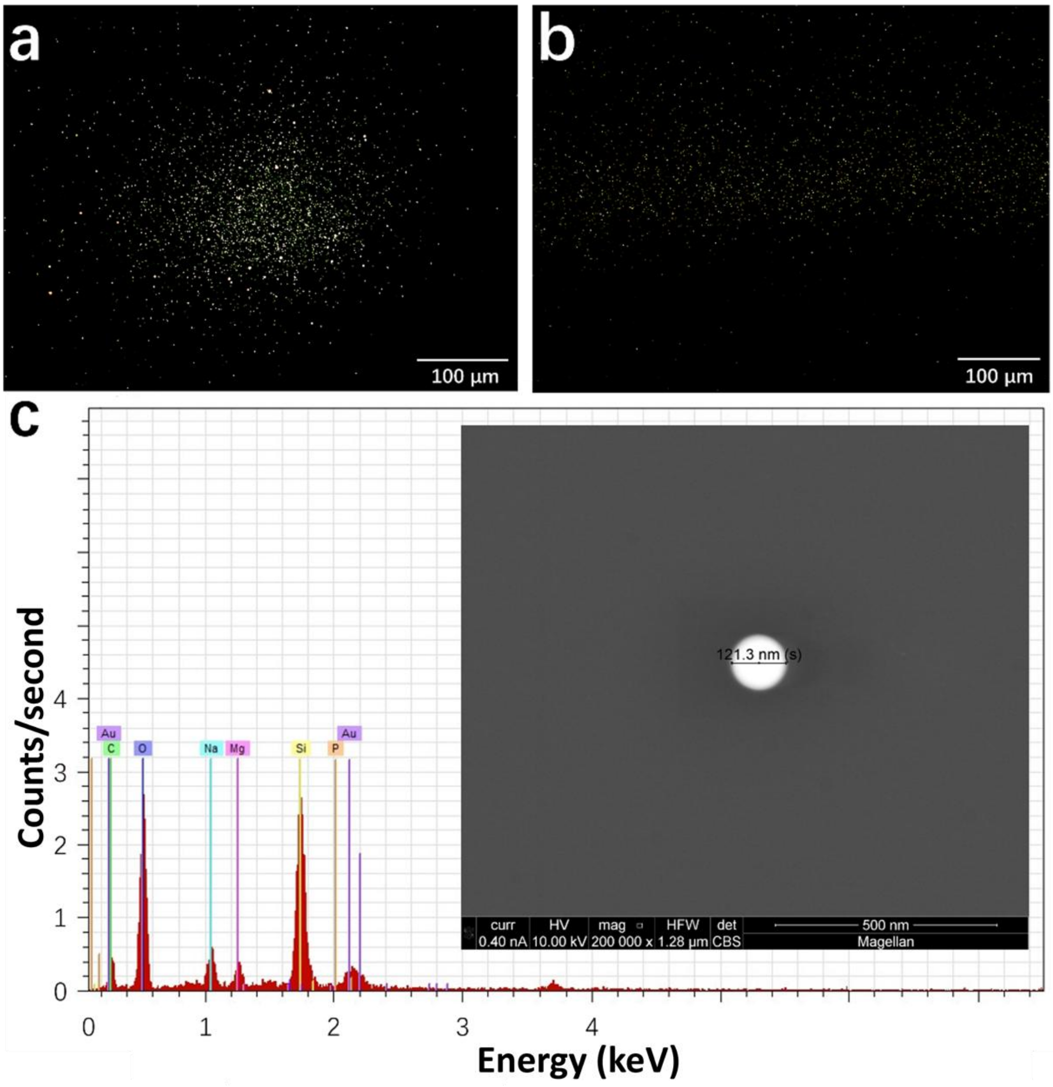

Figure S12. Dark-field optical images of the Au NPs photo-ejected from liquid and deposited on the bottom glass substrate in (a) point deposition and (b) line deposition experiments with most of the deposited NPs follow a Gaussian distribution as the laser intensity.[24] (c) Energy-dispersive X-ray (EDX) spectrum and SEM image (inserted figure) of an individual deposited Au NP.

SI9. Gaussian laser beam envelope calculation

To quantitatively investigate the envelope of Gaussian beam profile out of the liquid interface, we calculate the $1/e^2$ intensity radius ($w(z)$) of the laser beam using the following equation:

$$w(z) = w_0\sqrt{1 + (z/z_R)^2} \quad \text{(s16)}$$

where w_0 is the $1/e^2$ beam radius at the focal point, 6 μm, z is the beam propagating direction (**Figure 4c**) and z_R is the Rayleigh length ($z_R = w_0{}^2\pi/\lambda$, λ is the wavelength, 800 nm). The calculated $1/e^2$ intensity along the beam propagating direction out of the droplet/air interfaces is highlighted by the red solid lines as shown in **Figure 4c**. We note that the refractive index mismatch at the droplet/air interfaces cannot yield significant refraction of the beam envelope, as the beam spot size is much smaller than the size of the droplet.

SI10. The MD-simulated spread angles with respect to the direction normal to the liquid surface of 18 different NPs

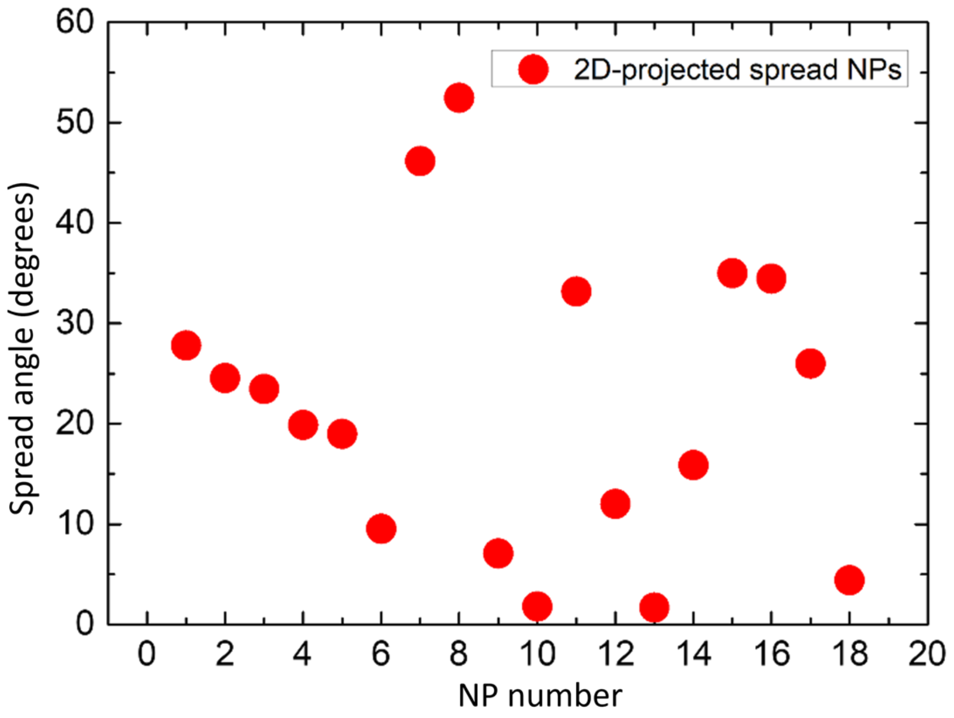

Figure S13. The MD-simulated spread angles of 18 different NPs.

SI11. The monolayer WSe_2 and photoluminescence spectroscopy characterization

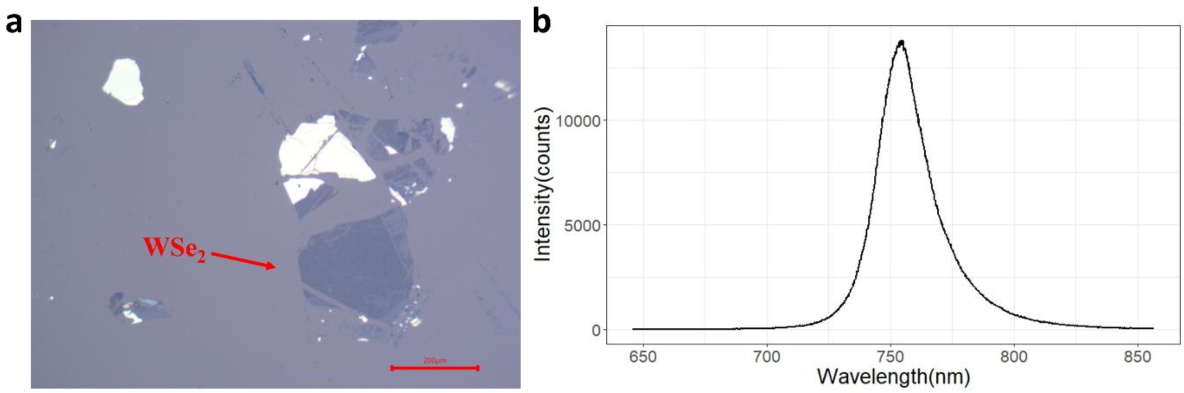

Figure S14. The bright-field optical image (a) and photoluminescence spectroscopy (b) of the fabricated monolayer WSe_2.

Supplementary references:

1. Guzmán, E. *et al.* A broad perspective to particle-laden fluid interfaces systems: from chemically homogeneous particles to active colloids. *Advances in Colloid and Interface Science* **302**, 102620 (2022).
2. Guzmán, E. *et al.* Particle-laden fluid/fluid interfaces: physico-chemical foundations. *J. Phys.: Condens. Matter* **33**, 333001 (2021).
3. Maestro, A., Guzmán, E., Ortega, F. & Rubio, R. G. Contact angle of micro- and nanoparticles at fluid interfaces. *Current Opinion in Colloid & Interface Science* **19**, 355–367 (2014).
4. Léandri, J. & Würger, A. Trapping energy of a spherical particle on a curved liquid interface. *Journal of Colloid and Interface Science* **405**, 249–255 (2013).
5. Guzmán, E., Ortega, F. & Rubio, R. G. Forces Controlling the Assembly of Particles at Fluid Interfaces. *Langmuir* **38**, 13313–13321 (2022).
6. Tang, Y. & Cheng, S. Capillary forces on a small particle at a liquid-vapor interface: Theory and simulation. *Phys. Rev. E* **98**, 032802 (2018).
7. Joanny, J. F. & de Gennes, P. G. A model for contact angle hysteresis. *J. Chem. Phys.* **81**, 552–562 (1984).
8. Lee, E., Huang, D. & Luo, T. Ballistic Supercavitating Nano Swimmer Driven by Single Gaussian Beam Optical Pushing and Pulling Forces. *Nat Commun* **11**, 2404 (2020).
9. Hinds, E. A. & Barnett, S. M. Momentum Exchange between Light and a Single Atom: Abraham or Minkowski? *Phys. Rev. Lett.* **102**, 050403 (2009).
10. Mansuripur, M. & Zakharian, A. R. Maxwell's macroscopic equations, the energy-momentum postulates, and the Lorentz law of force. *Phys. Rev. E* **79**, 026608 (2009).
11. Scalora, M. *et al.* Radiation pressure of light pulses and conservation of linear momentum in dispersive media. *Phys. Rev. E* **73**, 056604 (2006).

12. Ellingsen, S. Å. Theory of microdroplet and microbubble deformation by Gaussian laser beam. *J. Opt. Soc. Am. B, JOSAB* **30**, 1694–1710 (2013).

13. Nieminen, T. A. *et al.* Optical tweezers: Theory and modelling. *Journal of Quantitative Spectroscopy and Radiative Transfer* **146**, 59–80 (2014).

14. Preez-Wilkinson, N. du, Stilgoe, A. B., Alzaidi, T., Rubinsztein-Dunlop, H. & Nieminen, T. A. Forces due to pulsed beams in optical tweezers: linear effects. *Opt. Express, OE* **23**, 7190–7208 (2015).

15. Guo, G., Feng, T. & Xu, Y. Tunable optical pulling force mediated by resonant electromagnetic coupling. *Opt. Lett., OL* **43**, 4961–4964 (2018).

16. Lehmuskero, A., Johansson, P., Rubinsztein-Dunlop, H., Tong, L. & Käll, M. Laser Trapping of Colloidal Metal Nanoparticles. *ACS Nano* **9**, 3453–3469 (2015).

17. Metwally, K., Mensah, S. & Baffou, G. Fluence Threshold for Photothermal Bubble Generation Using Plasmonic Nanoparticles. *J. Phys. Chem. C* **119**, 28586–28596 (2015).

18. Baffou, G., Polleux, J., Rigneault, H. & Monneret, S. Super-Heating and Micro-Bubble Generation around Plasmonic Nanoparticles under cw Illumination. *J. Phys. Chem. C* **118**, 4890–4898 (2014).

19. Lukianova-Hleb, E. *et al.* Plasmonic Nanobubbles as Transient Vapor Nanobubbles Generated around Plasmonic Nanoparticles. *ACS Nano* **4**, 2109–2123 (2010).

20. Lee, E. & Luo, T. Long-distance optical pulling of nanoparticle in a low index cavity using a single plane wave. *Sci. Adv.* **6**, eaaz3646 (2020).

21. Lapotko, D. Optical excitation and detection of vapor bubbles around plasmonic nanoparticles. *Opt. Express, OE* **17**, 2538–2556 (2009).

22. Lachaine, R., Boulais, E., Bourbeau, E. & Meunier, M. Effect of pulse duration on plasmonic enhanced ultrafast laser-induced bubble generation in water. *Appl. Phys. A* **112**, 119–122 (2013).

23. Zhang, Q. *et al.* Surface Bubble Growth in Plasmonic Nanoparticle Suspension. *ACS Appl. Mater. Interfaces* **12**, 26680–26687 (2020).

24. Zhang, Q., Li, R., Lee, E. & Luo, T. Optically Driven Gold Nanoparticles Seed Surface Bubble Nucleation in Plasmonic Suspension. *Nano Lett.* **21**, 5485–5492 (2021).

25. Zhang, Q. *et al.* Light-Guided Surface Plasmonic Bubble Movement via Contact Line De-Pinning by In-Situ Deposited Plasmonic Nanoparticle Heating. *ACS Appl. Mater. Interfaces* **11**, 48525–48532 (2019).
